# Genome Evolution of Two Intertidal *Sargassum* Species (*S. fusiforme* and *S. thunbergii*) and Their Response to Abiotic Stressors

**DOI:** 10.1093/gbe/evaf084

**Published:** 2025-05-03

**Authors:** Hocheol Kim, Jihoon Jo, Ji Hyun Yang, Khaoula Ettahi, Yukyoung Jeon, Jundong Yu, Debashish Bhattacharya, Jong Hwan Kwak, Hwan Su Yoon

**Affiliations:** Department of Biological Sciences, Sungkyunkwan University, Suwon 16419, Korea; Department of Biological Sciences, Sungkyunkwan University, Suwon 16419, Korea; Division of Genetic Resources, Honam National Institute of Biological Resources, Mokpo 58762, Korea; Department of Biological Sciences, Sungkyunkwan University, Suwon 16419, Korea; Department of Biological Sciences, Sungkyunkwan University, Suwon 16419, Korea; School of Pharmacy, Sungkyunkwan University, Suwon 16419, Korea; Racing Laboratory, Korea Racing Authority, Gachon 13822, Korea; Department of Biochemistry and Microbiology, Rutgers University, New Brunswick, NJ 08901, USA; School of Pharmacy, Sungkyunkwan University, Suwon 16419, Korea; Department of Biological Sciences, Sungkyunkwan University, Suwon 16419, Korea

**Keywords:** *Sargassum thunbergii*, *Sargassum fusiforme*, genome evolution, desiccation stress, salicylic acid

## Abstract

*Sargassum fusiforme* and *Sargassum thunbergii* are ecologically and commercially important seaweeds that thrive in intertidal zones and are frequently exposed to extreme variation in environmental stress. Despite their importance, limited genomic information exists for these species, which hinders a comprehensive understanding of the evolution and adaptation of the genus *Sargassum* to marine coastal habitats. Two *Sargassum* genomes were generated in this study. The genome sizes of *S. fusiforme* and *S. thunbergii* were 438 and 376 Mbp, respectively, which are larger than the published genomes of the brown seaweed group, Ectocarpales. Expansion of the *Sargassum* genomes was significantly explained by the spread of transposable elements (TEs). Additionally, extensive gene duplications and their diversification occurred particularly through tandem, proximal, and dispersed duplications, which likely played an important role in response to environmental stress. Differentially expressed gene analysis under ambient and desiccation stress conditions confirmed that some duplicated genes respond to stress. We identified enhanced disease susceptibility 1 (EDS1) genes that promote salicylic acid (SA) biosynthesis, and their expansion is likely linked to TEs. We also confirmed the potential role of EDS1 by analyzing its subcellular localization (in *Arabidopsis thaliana*) and quantified the increased SA levels under desiccation conditions. This study demonstrates that the genomic evolution has played a critical role in allowing *S. fusiforme* and *S. thunbergii* to adapt to harsh intertidal conditions. The genomic resources of *Sargassum* species provided here will be instrumental in advancing future research, aiding in the understanding of adaptive evolution in brown algae.

**Significance**

*Sargassum* species are the most diverse group of brown algae, thriving in diverse marine ecosystems. Understanding their genomes is crucial to uncovering how they successfully adapt to harsh environments. However, only one *Sargassum* genome is currently available, still limiting insights into their evolution and adaptation. Our study fills this gap by providing de novo genome assemblies that highlights key adaptations of *Sargassum* to intertidal environments. We identified key genomic features such as transposable element expansion and gene duplication. We also confirmed the importance of salicylic acid synthesis in coping with environmental stress. Our results can provide a crucial resource enabling a better understanding of the evolution, adaptation, and ecological interactions of *Sargassum* species and even brown algal species.

## Introduction

Brown algae (Phaeophyceae) include around 2,100 species and have evolved complex traits that allow them to thrive in diverse environments, including the open ocean, coastal areas, and freshwater (Hakim and Patel 2020; Denoeud et al. 2024). Many brown algal species inhabit intertidal zones, where fluctuating tidal levels expose them to atmospheric conditions (Dittami et al. 2020; Hakim and Patel 2020). This frequent exposure makes them susceptible to both abiotic (e.g. desiccation, UV radiation, thermal stress, high light, nutrient limitation, and salinity fluctuation) and biotic stress (e.g. grazing, fouling, pathogens). In response to these challenges, brown algae have developed unique genome contents and metabolic processes (Cock et al. 2010; Dittami et al. 2020). Notably, alginate, a unique component constituting the brown algal cell wall, plays a significant role in responding to environmental stressors (Rabillé et al. 2019; Denoeud et al. 2024).

*Sargassum* C. Agardh is the most species-rich genus of Fucales in the Phaeophyceae. About 360 species are classified within the two main subgenera (i.e. *S.* subgen. *Bactrophycus* and *Sargassum*), based on morphology and phylogenetic analyses using several molecular markers (Mattio and Payri 2011; Dixon et al. 2014). *Sargassum* species are cosmopolitan, and their distribution includes tropical, subtropical, and temperate regions from intertidal to subtidal zones (Mattio and Payri 2009). Some species are pelagic, occasionally forming blooms and extensive mats on the ocean surface, i.e. the Sargasso Sea (Fidai et al. 2020). These floating mats provide habitats and nutrients for microbes, invertebrates, and fish (Gulick et al. 2023).

*Sargassum fusiforme* and *Sargassum thunbergii* have been the focus of much work due to their widespread use in the food and pharmaceutical industries. Their close evolutionary relationship is supported by phylogenetic analyses (Mattio and Payri 2011; Cho et al. 2012; Dixon et al. 2014). Both species are endemic to the Northwest Pacific and East Asia (Cho et al. 2012; Kobayashi et al. 2018). Because of their edible nature and utility, these species have been extensively cultivated in Korea, Japan, and China, particularly in the case of *S. fusiforme* (Hwang and Park 2020; Liu et al. 2021). These seaweeds have been extensively studied for their chemical compounds, including sulfated polysaccharides and polyphenols, involved in antioxidant, antiinflammatory, and antiviral activities (Luo et al. 2019; Rushdi et al. 2020).

In natural habitats, *S. fusiforme* and *S. thunbergii* proliferate on rocky shores as dominant species in coastal algal flora (Kang et al. 2011). These two *Sargassum* species frequently face several abiotic stresses, as do many species in intertidal zones. Multiple studies have demonstrated their robust tolerance to thermal, osmotic, salinity, and desiccation stress during germination (Chu et al. 2012; Zuo et al. 2023). When exposed to heat stress and UV radiation, these species undergo metabolic changes in amino acid synthesis and photosynthesis (Liu and Lin 2020; Sun et al. 2022). Carbon accumulation plays a crucial role during dehydration and rehydration, preventing cell membrane damage and regulating osmosis (Zhao et al. 2022). Furthermore, extracts from *Sargassum* species containing polyphenols inhibit UV-induced oxidative stress and inflammatory cytokine expression (Chen et al. 2022).

Salicylic acid (SA) is a phenolic acid and signaling molecule in seaweeds and plants that participates in metabolic pathways related to defense against abiotic stress and pathogens (Khan et al. 2015; Wani et al. 2017). SA plays a crucial role in regulating reactive oxygen species (ROS) levels induced by various environmental stressors and in modulating the redox control system (Saleem et al. 2021). SA is essential for plants to resist pathogens *via* transcriptional regulation of resistance genes and accumulation of other metabolites (Ding and Ding 2020). This polyphenol is also reported in brown algae, including *S. thunbergii* and *S. fusiforme* and is likely to be involved in enhancing stress resistance (Javed et al. 2025).

Recently, the genome of *S. fusiforme* from a Chinese population (hereafter referred to as *S. fusiforme* Ch.) was published (Wang et al. 2020). However, the relationship between genome content and response to abiotic stresses was not well elucidated in this study. Thus, comprehensive genome-wide information for these two species remains limited, making it challenging to understand the molecular traits involved in response to abiotic stress. In this study, we generated de novo assemblies of the genomes of *S. fusiforme* and *S. thunbergii* from Korean populations and analyzed differential gene expression and SA levels under desiccation stress to elucidate stress responses in these brown seaweeds.

## Results

### Genome Size Estimation and Genome Assemblies of *S. fusiforme* and *S. thunbergii*

Accurate genome size estimation in *Sargassum* species was challenging because these specimens were collected from natural habitats. Consequently, we could not determine the genome size of each species individually (supplementary figs. S1 and S2, Supplementary Material online). However, other approaches such as microscopic *C*-value measurement (Phillips et al. 2011) and the previous genome study of *S. fusiforme* Ch. (Wang et al. 2020) were important guidelines. Based on these results, we postulate that the genome sizes of both *Sargassum* species ranges from 196 to 394 Mb.

Long-read sequence data from the PacBio Sequel I platform were used to generate de novo genome assemblies of *S. fusiforme* and *S. thunbergii*. A total of 33.7 Gb of data from *S. fusiforme* and 96.7 Gb of data from *S. thunbergii* were generated in this study (supplementary table S1, Supplementary Material online). Despite this discrepancy in the amount of the generated data for the two *Sargassum* species resulting from the complex polysaccharides that hindered DNA extraction, the coverages of long-read data were 84 × and 241× of the expected genome sizes, which exceeded the recommended depth (40× —60×) for de novo genome assembly (Jung et al. 2020).

We used Falcon to assemble the data (Chin et al. 2016). The EukRep classification tool was used to remove prokaryotic contigs from the initial assembly (West et al. 2018). Additionally, we thoroughly verified the assembled genomes. We examined 18S sequences to check for potential contamination from other eukaryotes and confirmed that all predicted genes showed top matches with brown algal species. About 438 Mb of *S. fusiforme* genome was finally assembled. The number of contigs in the *S. fusiforme* genome assembly was 1,576, the N50 was 440,341 bp, and GC content was 48.45% (Table 1). The new *S. fusiforme* genome assembly was 43.7 Mb larger and exhibited greater contiguity (1,576 contigs < 6,750 contigs) and completeness (79.2% > 76.9%, complete BUSCOs in genome mode) when compared with the existing *S. fusiforme* Ch. genome assembly (Table 1 and supplementary fig. S3, Supplementary material online), suggesting that it is of higher quality (Wang et al. 2020). For *S. thunbergii*, a genome of size 376 Mb was assembled. The number of contigs was 2,347, the N50 was 244,659 bp, and GC content was 48.63% (Table 1).

**Table 1:** Summary of features of the assembled *S. thunbergii* and *S. fusiforme* genomes and comparison to the previously reported *S. fusiforme* genome (Wang et al. 2020)

	*S. thunbergii* Kr. (This study)	*S. fusiforme* Kr. (This study)	*S. fusiforme* Ch. (Wang et al. 2020)
Genome size (bp)	376,248,926	438,130,216	394,427,661
Number of contigs	2,347	1,576	6,750
N50 (bp)	244,659	440,341	142,085
L50	465	292	775
Largest contigs size (bp)	1,605,781	3,450,153	931,042
GC (%)	48.63	48.45	48.41
Number of genes	19,210	21,691	20,222
Complete BUSCOs	214 (83.9%)	212 (83.2%)	214 (83.9%)
Complete and single-copy BUSCOs	204 (80.0%)	197 (77.3%)	204 (80.0%)
Complete and duplicated BUSCOs	10 (3.9%)	15 (5.9%)	10 (3.9%)
Fragmented BUSCOs	16 (6.3%)	28 (11.0%)	23 (9.0%)
Missing BUSCOs	25 (9.8%)	15 (5.8%)	18 (7.1%)

### Genome Size Expansion and Repeat Elements in *Sargassum* Genomes

**Fig. 1.**
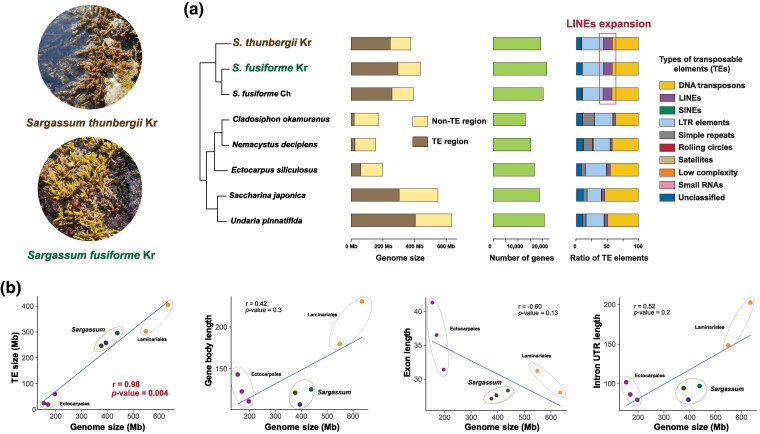
Genome assembly results and genomic content of *S. thunbergii* and *S. fusiforme*. a) Genome features including genome size, the number of genes, and TE element ratio of *S. thunbergii* and *S. fusiforme* compared with the published Ectocarpales and Laminariales genomes. These results suggest significant LINE expansion occurred in the two *Sargassum* genomes. b) Contribution of genome features to the expansion of the two *Sargassum* genomes, emphasizing the significant role played by TEs in genome expansion when compared with gene body, exon, and intron + UTR lengths. The correlation coefficient (*r*) from the Spearman correlation analysis is presented in each plot along with the corresponding *P*-value.

The two *Sargassum* assemblies were compared to six previously published genomes of brown algae (Fig. 1a). Overall genome sizes of the *Sargassum* species including *S. fusiforme* Ch. were larger than those of *Cladosiphon okamuranus* (Nishitsuji et al. 2016), *Nemacystus decipiens* (Nishitsuji et al. 2019), and *Ectocarpus siliculosus* (Cock et al. 2010) in Ectocarpales and smaller than those of *Saccharina japonica* (Ye et al. 2015) and *Undaria pinnatifida* (Graf et al. 2021) in Laminariales.

*Sargassum* genome size expansion was significantly attributed to transposable element (TE) expansion. In particular, long interspersed nuclear elements (LINEs) exhibited a distinct increase in *Sargassum* species (Fig. 1a). The *S. fusiforme* genome contained about 42.3 Mbp of LINEs, whereas the *S. thunbergii* genome contained around 35.7 Mbp of LINEs (supplementary fig. S4 and supplementary table S2, Supplementary Material online). To investigate the main factor contributing to the expansion in *Sargassum* genomes, we examined the correlation between genome size and several genomic factors such as TE size, length of genes, exon length, and intron length. These results confirmed that the increase in genome size in *Sargassum* is primarily explained by TE insertions (Fig. 1b).

### Gene Prediction and Gene Content Comparison of *Sargassum* Genomes

About 21,691 genes were predicted in *S. fusiforme*, which was a bit more than in the *S. fusiforme* Ch. (i.e. 20,222 in Wang et al. 2020). About 19,210 genes were predicted in the *S. thunbergii* genome. The completeness of gene models was assessed using BUSCO scores (protein mode), with ∼83.2% completeness observed in the *S. fusiforme* genome, whereas the value was about 83.9% for *S. thunbergii* (Table 1 and supplementary table S3, Supplementary Material online). Predicted genes that were functionally annotated using five different approaches numbered 17,875 (82.4% of predicted genes) and 16,236 genes (84.5% of predicted genes) for the *S. fusiforme* and *S. thunbergii*, respectively (supplementary fig. S5, Supplementary Material online).

We compared the predicted genes of *S. thunbergii*, *S. fusiforme*, and *S. fusiforme* Ch. genomes using Orthovenn3 (Sun et al. 2023). A total of 14,796 clusters were identified, and 10,707 were shared in all of the studied *Sargassum* genomes (supplementary fig. S6, Supplementary Material online). To explore the functions of the nonshared genes in three genomes, Gene Ontology (GO) terms and their enrichment patterns were analyzed. The enriched GO terms included ubiquitin-dependent protein catabolic processes, DNA integration, peroxidase activity, ammonium transmembrane transporter activity, and nucleosome assembly (supplementary table S4, Supplementary Material online).

Although differences in gene contents exist, assessing their impact on physiological variation in *Sargassum* remains challenging. Three *Sargassum* genomes have conserved core gene functions, and the lack of genome completeness may lead to overestimating observed genetic differences because of gene splitting in highly fragmented assemblies despite high BUSCO scores in *Sargassum* genomes (Denton et al. 2014). Therefore, we focused on identifying genomic features that are shared among *Sargassum* species and distinguish them from other brown algae, rather than analyzing *Sargassum* genome variation itself in the following sections.

### Evolution of Gene Families and Gene Duplication in *Sargassum* Genomes

**Fig. 2.**
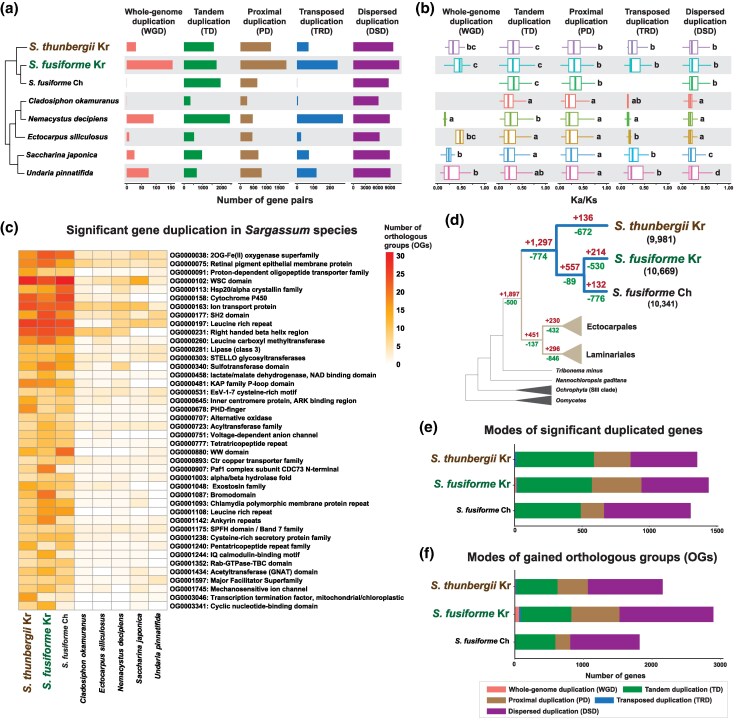
Gene duplications and their different modes in *Sargassum* genomes. a) Number of duplicated genes in *Sargassum* and brown algal genomes. b) *K*_a_/*K*_s_ ratio of duplicated genes in different modes in *Sargassum* and brown algal genomes. Since the data did not follow a normal distribution, the Kruskal–Wallis H test was performed to compare *K*_a_/*K*_s_ ratios among species, revealing significant difference (*P* < 0.01). Dunn's test was used for post hoc pairwise comparisons with different alphabet letters indicating statistically significant differences between groups. c) Orthogroups with highly duplicated genes in *Sargassum* genomes compared with other brown algal genomes. d) Results of gene gain and loss analyses based on orthologue comparisons. e) Duplication modes of significantly duplicated genes in *Sargassum* genomes. f) Duplication modes of the gained OGs in *Sargassum*.

Gene duplication has played a major role in *Sargassum* genome evolution. 12,636 genes in *S. thunbergii* and 15,155 genes in *S. fusiforme* genomes were classified as duplicated genes. To further understand the evolutionary processes that generated these gene families, we analyzed gene duplication modes and their *K*_a_/*K*_s_ ratios following Qiao et al (2019). In *S. thunbergii*, we found evidence for 31 whole-genome duplications (WGD), 1,587 tandem duplications (TD), 1,171 proximal duplications (PD), 69 transposed duplications (TRD), and 9,778 dispersed duplications (DSD). While 156 WGD, 1,733 TD, 1,765 PD, 246 TRD, and 11,255 DSD were classified in *S. fusiforme*, WGD and TRD were not detected in *S. fusiforme* Ch., which was likely due to the highly fragmented genome, although the exact reason remains unclear. Nonetheless, the overall number of gene duplications in *Sargassum* was higher than in other brown algae. *Sargassum* species exhibited approximately twice as many duplications as *C. okamuranus* and *E. siliculosus* (Fig. 2a and supplementary table S5, Supplementary Material online).

The distributions of *K*_a_/*K*_s_, *K*_a_ (nonsynonymous substitutions), and *K*_s_ (synonymous substitutions) across different gene duplication modes were investigated. Overall, duplicated genes in *Sargassum* exhibited high *K*_a_/*K*_s_ ratios (0.262 ± 0.136). In particular, *K*_a_/*K*_s_ ratios for TD and PD gene pairs in *Sargassum* species (TD: 0.319 ± 0.156; PD: 0.323 ± 0.158) were higher than those in other brown algal species (TD: 0.246 ± 0.151; PD: 0.256 ± 0.159). Whereas the *K*_a_/*K*_s_ ratios of TRD and DSD in *Sargassum* (TRD: 0.286 ± 0.144; DSD: 0.249 ± 0.128) were similar to those in Laminariales species (TRD: 0.282 ± 0.157; DSD: 0.229 ± 0.128), they were higher than in Ectocarpales species (TRD: 0.156 ± 0.020; DSD: 0.166 ± 0.054, Fig. 2b and supplementary table S6, Supplementary Material online). Moreover, *K*_a_ (0.440 ± 0.321) and *K*_s_ (2.15 ± 1.75) values of duplicated genes were relatively low (supplementary fig. S7 and supplementary table S6, Supplementary Material online). In particular, the distinctly lower *K*_a_ and *K*_s_ values of TD and PD in *Sargassum* genomes (*K*_a_ of TD: 0.180 ± 0.153, *K*_s_ of TD: 0.560 ± 0.517, *K*_a_ of PD: 0.203 ± 0.162, *K*_s_ of PD: 0.587 ± 0.505) were calculated in comparison with other brown algal species (*K*_a_ of TD: 0.246 ± 0.189, *K*_s_ of TD: 1.43 ±1.29, *K*_a_ of PD: 0.317 ± 0.214, *K*_s_ of PD: 1.76 ± 1.40).

Among duplication genes, we identified orthogroups (OGs) in the *Sargassum* genomes with highly duplicated genes, when compared with other brown algae (Fig. 2c and supplementary table S7, Supplementary Material online). These genes were primarily duplicated through three modes: TD, PD, and DSD (Fig. 2e), and the majority were classified as having functions in posttranslational modification, protein turnover, and chaperones within Clusters of Orthologous Groups (COG) categories (supplementary fig. S8, Supplementary Material online).

Gene gains and losses were calculated using Dollo parsimony (Csűös 2010). A high number of gene gains and losses (+1,297 OGs, −774 OGs) were found at the node of the ancestor of *Sargassum*, when compared with the more modest numbers at the nodes uniting Ectocarpales (+230 OGs, −432 OGs) and Laminariales (+296 OGs, −846 OGs). This suggests that the *Sargassum* lineage gained more novel genes during their evolution than did the two sister lineages (Fig. 2d and supplementary fig. S9and supplementary table S8, Supplementary Material online). In *Sargassum*, TD, PD, and DSD primarily contributed to the gain/loss process (Fig. 2f). In these species, genes associated with intracellular trafficking, secretion, vesicular transport, and transcription were predominantly gained and genes related to lipid transport and metabolism were lost (supplementary fig. S10, Supplementary Material online).

### Differentially Expressed Genes Against Desiccation Stress

**Fig. 3.**
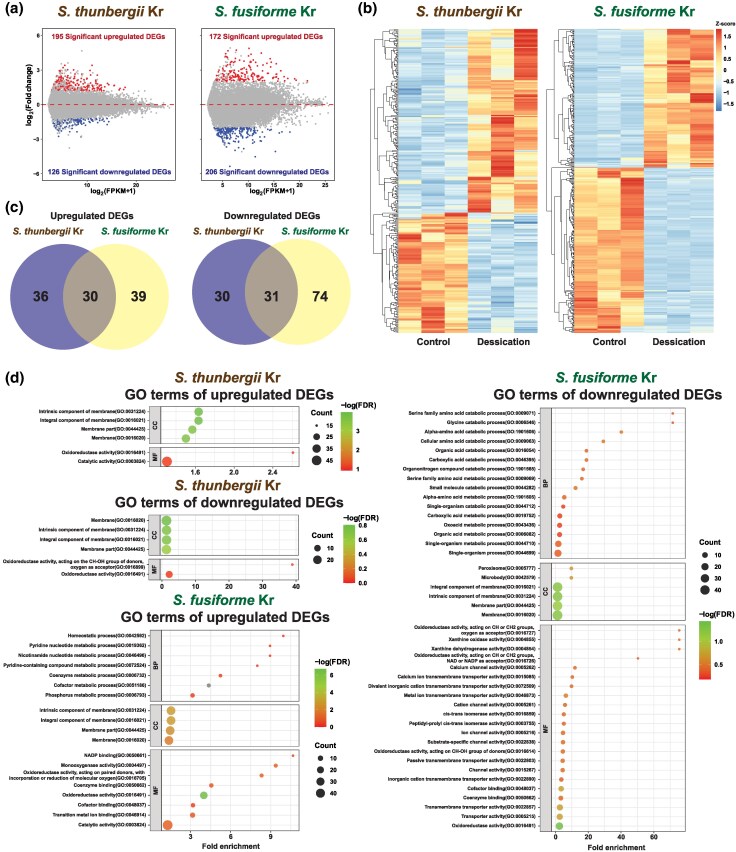
Differentially expressed gene analysis of *S. thunbergii* and *S. fusiforme* between control and desiccation stress. a) Minus average plots showing log_2_(fold change) versus log_2_(FPKM+1) plots and the number of upregulated and downregulated DEGs. b) Heatmap of DEGs. c) Shared and divergent functions of the upregulated and downregulated DEGs between *S. thunbergii* and *S. fusiforme*. d) GO terms of the upregulated and downregulated DEGs.

To examine the transcriptional response of *Sargassum* species to desiccation stress, we generated transcriptome data from sporophyte tissue. Based on the differentially expressed gene (DEG) analysis from these transcriptomes, we identified 195 upregulated genes and 126 downregulated genes in *S. thunbergii*, whereas 172 upregulated genes and 206 downregulated genes were found in *S. fusiforme* (Fig. 3a and b and supplementary table S9, Supplementary Material online). There were 30 genes with analogous functions among the upregulated DEGs in both species and 31 genes with similar functions among the downregulated DEGs (Fig. 3c). Upregulated and downregulated DEGs in both species showed significant enrichment of membrane-related GO terms and oxidoreductase-related terms. The DEGs of *S. fusiforme,* which had more significant GO terms than *S. thunbergii*, were enriched in various metabolic processes such as peroxisome, microbody, ion transmembrane transporter activities, and ion channel activities (Fig. 3d).

### Transcriptional Expression of Duplicated Genes Against Desiccation Stress

**Fig. 4.**
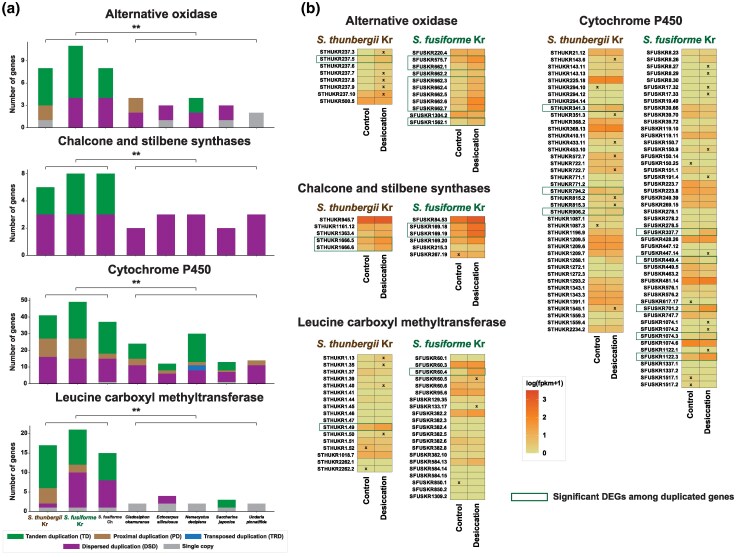
Duplication modes and expression pattern of four key gene families associated with abiotic stress including AOX, CHS and STS synthases, CYPs, and LCMT. a) Duplication modes of four gene families. Due to unequal variances between the *Sargassum* group and other brown algal groups, Welch's *t*-test was conducted for the comparison. The asterisk mark (**) indicates *P* < 0.01. b) Mean gene expression profiles of four gene families across triplicate under control and desiccation conditions. x marks indicate the presence of an outlier among the triplicates. Genes detected as significant DEGs are marked with boxes.

Gene duplication underpins adaptation to changing environments (Kondrashov 2012; Mattenberger et al. 2017). Among the duplicated genes in *Sargassum*, which were more abundant than in other brown algae (Fig. 2c), we identified some that were significantly differentially expressed (Fig. 4). These include four key gene families that have a strong association with abiotic stress responses in plants: alternative oxidase (AOX), chalcone (CHS) and stilbene (STS) synthase, cytochrome P450 (CYP), and leucine carboxyl methyltransferase (LCMT).

AOX acts in the mitochondrial electron transport chain and improves photosynthetic efficiency under osmotic and temperature stress (Saha et al. 2016). CHS and STS are critical in flavonoid and stilbenoid biosynthesis, which provide defense against UV-light damage and pathogens (Vannozzi et al. 2012). CYP participates in plant development and defense against environmental stressors (Xu et al. 2015). LCMT plays roles in methylating carboxylic acids, with specific stress-related functions in plants including rice (Seo and Jang 2023).

In *S. thunbergii*, five AOX genes were duplicated via TD and two via PD, while one gene was a single copy. In *S. fusiforme*, seven AOX genes were duplicated through TD and four through DSD. For CHS and STS genes, two TD and three DSD were identified in *S. thunbergii*, whereas three TD and three DSD events occurred in *S. fusiforme*. Regarding CYP genes, *S. thunbergii* exhibited 14 TD, 11 PD, and 16 DSD, while *S. fusiforme* showed 22 TD, 12 PD, and 15 DSD. LCMT gene duplications in *S. thunbergii* included 11 TD, four PD, one DSD, and one single-copy gene, whereas nine TD, two PD, nine DSD, and one single-copy gene were identified in *S. fusiforme* (Fig. 4a). All these duplicated gene numbers in *Sargassum* were significantly higher than in other brown algae (*P* < 0.01).

The expression pattern of these duplicated genes including DEGs appeared to be responsive to desiccation stress. Among the duplicated genes, one AOX gene *in S. thunbergii* and eight in *S. fusiforme* were identified as significant DEGs. Additionally, two CHS and STS genes from each species exhibited DEGs. Three CYP genes in *S. thunbergii* and five CYP genes in *S. fusiforme* were differentially expressed. Finally, one LCMT gene from each species showed significantly different expression levels (Fig. 4b).

### Discovery of the Genes Related to SA Synthesis Pathway

**Fig. 5.**
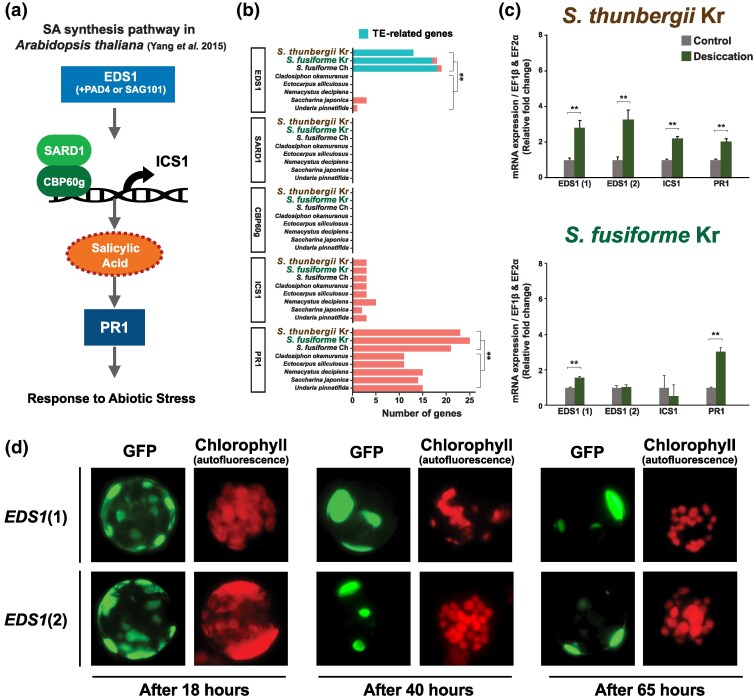
Discovery core genes involved in SA synthesis. a) Reference used to search for SA pathway genes. b) Number of SA synthesis pathway genes in *Sargassum* and brown algal genomes. SARD1 and CBP60g were not detected, and the number of ICS1 was not significantly different across genomes. However, the number of EDS1 and PR1 was significantly higher in *Sargassum* genomes compared with other brown algal genomes (*P* < 0.01, Welch's *t*-test). Additionally, most EDS1 genes in *Sargassum* were related to TEs. c) qPCR results of SA synthesis pathway genes based on the desiccation treatment. d) Results of subcellular localization analysis using *Arabidopsis* protoplasts to infer the localization *S. thunbergii* EDS1.

Even though SA is a major signaling molecule involved in stress responses to abiotic stress in plants and seaweeds (Khan et al. 2015; Wani et al. 2017), genome-wide profiling of this pathway has not yet been investigated in brown algae. To address this gap, we investigated the domain composition of the representative SA pathway in *Sargassum* and five brown algal genomes (supplementary table S10, Supplementary Material online). We referred to Yang et al. (2015) for a brief SA synthesis pathway in *Arabidopsis* as a guide (Fig. 5a).

The key regulator of the SA pathway in plants, enhanced disease susceptibility 1 (EDS1), was highly duplicated in the *Sargassum* genomes (13 copies in *S. thunbergii*, 18 copies in *S. fusiforme*, and 19 copies in *S. fusiforme* Ch.), but absent in Ectocarpales and only few copies exist in Laminariales species (three copies in *S. japonica* and one copy in *U. pinnatifida*). This increase in EDS1 copy number in *Sargassum* was likely associated with TEs because most of these gene sequences overlapped with TEs (Fig. 5b). For this reason, multiple copies of EDS1 were discovered in the repeat-unmasked gene models in both species. A similar case was previously observed in a study investigating resistance genes in Brassicaceae, indicating the importance of considering repetitive regions in brown algal genomes for accurate gene prediction (Slotkin 2018).

We also identified isochorismate synthase 1 (ICS1) gene and pathogenesis-related gene (PR1) in *Sargassum*. The number of PR1 genes in these species (21 to 25 copies) was markedly higher than in other brown algal genomes (11 to 15 copies). However, transcription factors such as SARD1 and CBP60 g, which are essential in plant SA signaling, were not detected in all brown algal genomes (Fig. 5b). To verify whether the identified genes involved in the SA synthesis pathway are responsive to desiccation stress, we examined their expression levels using qPCR. One representative gene from the duplicated genes of EDS1, ICS1, and PR1 was selected (supplementary table S11, Supplementary Material online). The qPCR results confirmed that these genes are transcriptionally active under desiccation stress conditions (Fig. 5c).

### Putative EDS1 Subcellular Localization

EDS1 subcellular localization was identified to gain insights into its potential function because this protein participates in SA signal transduction and regulates defense genes under environmental stress (Venugopal et al. 2009; Bernacki et al. 2018). We used EDS1 information from *S. thunbergii* to design the subcellular localization experiment (supplementary table S12, Supplementary Material online). Two *S. thunbergii* EDS1 homologs coupled to a green fluorescent protein (GFP) were transiently expressed in *Arabidopsis* protoplasts to determine their localization. Data were collected after 18, 40, and 64 h. These proteins were localized to the endomembrane in numerous vesicle-like structures after 18 h, which gathered and faded after 40 and 64 h (Fig. 5d). This indicates that EDS1 is present in prevacuolar compartments (PVCs) or multivesicular bodies/late endosomes (MVB/LE).

#### Identification and Quantification of SA in *Sargassum*

**Fig. 6.**
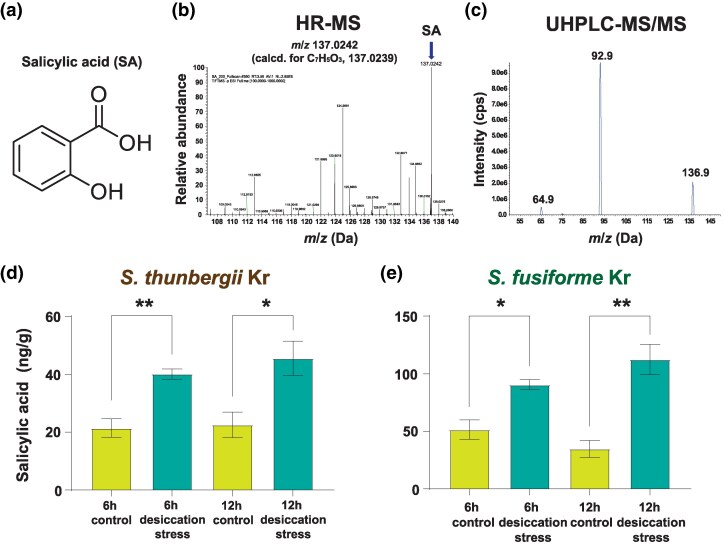
Qualification and quantification of SA in two *sargassum* species. a) Chemical structure of SA. b) High-resolution mass spectrum of SA from an UHPLC/Q Oribitrap High Resolution Mass Spectrometer in a negative ion mode. c) Product ion scan chromatogram of SA. UHPLC–MS/MS analysis was performed via gradient elution on an ACQUITY UPLC HSS C18 100 Å 1.8 µm column (2.1 × 150 mm). A triple quadrupole mass spectrometer was operated in negative ionization mode, and the separated analytes were detected using MRM mode at *m/z* 136.9 → 92.9 for the quantification and *m/z* 136.9 → 65.1 for the qualification of SA. d) Content of SA in control and desiccation stress groups (6 and 12 h) of *S. thunbergii.* e) Content of SA in control and desiccation stress groups (6 and 12 h) of *S. fusiforme.* Two-sample *t*-tests were conducted because the control and desiccation stress groups showed equal variances in the experiments. Asterisk marks indicate significance (*P < 0.05 and **P < 0.01).

Salicylic acid (Fig. 6a) was extracted from *S. thunbergii* and *S. fusiforme* to investigate its role and production under desiccation conditions. We established methods for SA quantification (described in supplementary fig. S11 and supplementary table S13, Supplementary Material online) and compared SA content between two *Sargassum* species following 6 and 12 h of desiccation. SA was detected using high-resolution mass spectrometry (HR-MS; Fig. 6b) followed by ultra-high-performance liquid chromatography–tandem mass spectrometry (UHPLC–MS/MS). UHPLC–MS/MS, known for its high selectivity and sensitivity, was employed to generate more precise measurements (Rodriguez-Aller et al. 2013). The analytes were detected and quantified based on the transition sets for SA (m/z 136.9 → 92.9 and 136.9 → 65.1) and for the internal standard (IS) (m/z 205 → 159 and 205 → 161) using multiple reaction monitoring (MRM) mode (Fig. 6c and supplementary fig. S12, Supplementary Material online). Based on this approach, we verified that *S. thunbergii* showed a significant increase in SA content after 6 and 12 h under desiccation stress when compared with control groups, increasing from 21.39 ± 3.21 to 40.08 ± 1.77 ng/g after 6 h and from 22.49 ± 4.43 to 45.50 ± 5.89 ng/g after 12 h (Fig. 6d). Similarly, *S. fusiforme* exhibited substantial increases in SA content under desiccation stress, rising from 51.41 ± 8.45 to 90.48 ± 4.41 ng/g after 6 h and from 34.75 ± 7.45 to 112.35 ± 13.28 ng/g after 12 h (Fig. 6e and supplementary fig. S13 and supplementary table S14, Supplementary Material online).

## Discussion

Many brown algae, including *Sargassum* species, thrive in harsh intertidal and coastal environments by evolving diverse mechanisms to deal with environmental stress. Genomic information is crucial for understanding these adaptations, but related studies and available genomes have been limited thus far. Recently, the Phaeoexplorer consortium (Denoeud et al. 2024) released a collection of brown seaweed genome assemblies, which revealed that the acquisition of genes linked to alginate and halogen synthesis contributed to robust cell walls and adhesion to substrates, aiding survival in dynamic coastal environments. However, the quality of some of these genomes, notably those of Fucales (including *Sargassum*), was lower (supplementary table S15, Supplementary Material online; Denoeud et al. 2024). Compared with these previous results, our *Sargassum* genome assemblies show a marked improvement.

Our study reveals that *S. fusiforme* and *S. thunbergii* have evolved several genomic and physiological features that likely play central roles in environmental adaptation. One key genomic component is TEs (Fig. 1b), which are repetitive DNA sequences capable of self-replication and reinsertion within a genome and can act as important drivers of species diversification (Ricci et al. 2018; Carducci et al. 2019). In brown algae, TEs are thought to play a significant role in genome evolution. Among TEs, long-terminal repeats have been identified as major contributors to genome expansion in brown algae (Cock et al. 2010, Ye et al. 2015).

Several studies suggest that TE-induced mutations and subsequent alterations in transcriptional regulation are associated with responses to abiotic stress (Belyayev 2014; Makarevitch et al. 2015). The observed expansion of TEs in the *Sargassum* genomes likely represents an adaptive mechanism in response to abiotic stress. In particular, a potential link between EDS1 genes and TEs was observed in our study (Fig. 5b). The significant role of TEs in coping with abiotic stress was highlighted by examining transcriptional responses under desiccation stress. However, similar studies in other brown algae remain scarce, which makes it hard to understand the overall relationship between TE expansion and adaptation in brown algae. Thus, more research should be done to explore the role of TEs in brown algae under various stress conditions.

Gene duplication and divergence of gene family members are key drivers in the evolution of brown algae. The expansion of gene families involved in carbohydrate metabolism, signaling pathways, and transcriptional regulation may have contributed to enhanced multicellular complexity and cell–cell communication in these taxa (Denoeud et al. 2024). Additionally, the amplification of genes related to halogen concentration, development, and defense mechanisms has enabled brown algae to successfully thrive in diverse marine environments having different environmental stressors (Cock et al. 2010, Ye et al. 2015, Denoeud et al. 2024). Our results also indicated that overall gene duplication and the expansion of gene families were related to signal pathways and transport systems (supplementary figs. S8 and S10, Supplementary Material online), which enabled *Sargassum* to successfully adapt by coping with environmental stress.

In the *Sargassum* genomes, TD, PD, and DSD were identified as the predominant modes of duplication (Fig. 2). Among these, TD and PD have been particularly important for the evolution of stress responses in *Sargassum*. TD and PD are crucial duplication modes for plant adaptation by expanding gene families involved in stress responses and functional diversification. Such mechanisms enhance plant resilience in challenging environments. For example, in Solanaceae species, TD was highly associated with disease resistance and stress adaptation (Huang et al. 2022). Interestingly, TD and PD genes in *Sargassum* exhibited high *K*_a_/*K*_s_ ratios but low *K*_a_ and *K*_s_ values (supplementary fig. S7 and supplementary table S6, Supplementary Material online), indicating that these duplications occurred relatively recently, but underwent rapid diversification under strong positive selection, which played an important role in effective adaptation.

A similar situation holds for genes in *Sargassum* that likely respond to abiotic stress. When exposed to air and water loss during low tide, *Sargassum* faces desiccation stress including oxidative stress, UV radiation, temperature fluctuations, and nutrient scarcity, triggering various physiological responses (Flores-Molina et al. 2014). These responses include mechanisms to reduce ROS levels and the activation of rehydration systems to prevent damage and maintain the integrity of cellular structures (Contreras-Porcia et al. 2011, 2023). Our DEG analysis under desiccation stress reveals oxidoreductase and ion channel activities (GO terms), suggesting that several genes related to ROS and dehydration actively contribute to stress abatement in *Sargassum* (Fig. 3d). Moreover, we found that duplicated genes that alleviate abiotic stress in plants also actively respond to desiccation stress in *Sargassum* (Fig. 4).

We demonstrated that SA plays a critical role in the response of *Sargassum* to abiotic stress (Fig. 5), with quantitative analyses indicating that SA levels increase under desiccation stress (Fig. 6). We observed a significant duplication of EDS1 genes in *Sargassum* genomes, potentially mediated by TEs (Fig. 5b). Since EDS1 promotes SA accumulation (Yang et al. 2015, Wani et al. 2017), the expansion of EDS1 genes may enhance *Sargassum* species to cope with environmental stress more effectively. Additionally, the transcription factors SARD1 and CBP60g, which bind to the promoter of ICS1 and activate its expression in plants (Yang et al. 2015), are absent in *Sargassum* genomes (Fig. 5b). This suggests that these plant-specific transcription factors may have been replaced by alternative regulatory genes in the brown algal lineage (Qin et al. 2018). However, transcription factors in *Sargassum* have not yet been characterized, and further research is needed to elucidate the SA regulatory pathway in *Sargassum*.

Subcellular localization analysis revealed that EDS1 is present in PVCs or multivesicular bodies/late endosomes (MVB/LE, Fig. 5d). PVC/MVBs are membrane-bound organelles that mediate protein trafficking from Golgi or the plasma membrane to vacuoles and participate in inactivated protein degradation (Cui et al. 2016). Protein trafficking to vacuoles, including protein storage vacuoles and lytic vacuoles , is a crucial process because vacuoles play a vital role in defense against stressors such as the accumulation of toxic molecules and the maintenance of turgor pressure (Sampaio et al. 2022). Thus, our findings suggest that EDS1, mediated by PVCs and MVBs, contributes to stress alleviation by influencing protein degradation and signaling pathways.

Our results indicate that *S. fusiforme* and *S. thunbergii* have evolved distinct genomic traits that enable them to thrive in marine environments. *Sargassum thunbergii* predominantly inhabits the upper intertidal zone, whereas *S. fusiforme* is found in the lower intertidal zone (Kang et al. 2011). Based on this observation, we hypothesized that *S. thunbergii* may have evolved more specialized adaptations to cope with environmental stress than *S. fusiforme*. However, our study demonstrates that both species share similar responses to environmental stress through mechanisms such as TE insertion and gene duplication.

In addition, we confirmed that genes involved in SA synthesis are active in both species, and the increase in SA level plays a role in mitigating desiccation stress. Thus, these findings suggest that both close-related *Sargassum* species likely share common mechanisms for alleviating environmental stress that were inherited from a common ancestor (Mattio and Payri 2011; Dixon et al. 2014). Nonetheless, the genomes of other *Sargassum* species are yet to be sequenced and the functions of numerous genes in the *Sargassum* genomes remain unknown. Therefore, future research should focus on sequencing additional *Sargassum* genomes and characterizing unstudied genes to deepen our understanding of *Sargassum* evolution and their adaptation to marine environments.

## Materials and Methods

### *Sargassum* Collection, Genomic DNA Extraction, and Sequencing

*Sargassum thunbergii* and *S. fusiforme* were collected from rocky shores at Anin Beach, Gangneung-si, Republic of Korea (37°44′04.7″N 128°59′24.7″E). After collection, the samples were brought to the laboratory. Any contaminants, such as small barnacles and parasitic algae, were removed using forceps and brushes. The samples were cleaned with sterile seawater several times and stored at −80 °C before genomic DNA isolation. DNA extraction was carried out based on the modified a CTAB-based extraction procedure using a high concentration of NaCl to remove polysaccharides and PVP to eliminate polyphenols in the extraction buffer during DNA purification.

The isolated genomic DNA was sent to DNA-link Inc., Seoul, Republic of Korea. For *S. thunbergii*, two SMRT cells of PacBio Sequel I (Pacific Biosciences, Inc., Menlo Park, CA, USA) were produced, while one cell was generated for *S. fusiforme* (supplementary table S1, Supplementary Material online). Short-read sequencing libraries were prepared using the Illumina TruSeq DNA nano kit, and sequences were generated using NovaSeq 6000 platform (Illumina, San Diego, USA; supplementary table S16, Supplementary Material online).

### Genome Size Estimation and Assembly

The genome sizes of two *Sargassum* species were estimated based on k-mer frequency distribution by using short-read data and Jellyfish v2.2.10 (Marçais and Kingsford 2011). As many bacterial contaminants were expected to be included in the data, bacteria and other contaminants reads were eliminated using BlobTools v1.1 (Laetsch and Blaxter 2017) before genome size estimation. k-mer count histograms were visualized using GenomeScope 2.0 (Ranallo-Benavidez et al. 2020).

The whole-genome assembly of two species was conducted using Falcon assembler (Chin et al. 2016). The assembled genomes were polished Falcon-Unzip (Chin et al. 2016). Following the assembly step, prokaryotic contigs were identified by EukRep v0.6.6 (West et al. 2018) and subsequently removed. The removed contigs were double-checked using BlastN (Camacho et al. 2009) search against the NCBI bacteria database. Furthermore, excessively short contigs (<25,000 bp) were eliminated. The filtered genomes underwent another polishing step using Pilon v1.22 (Walker et al. 2014) with the Illumina reads being mapped to the genomes. Before use, short reads were first trimmed using Trimmomatic v0.39 (Bolger et al. 2014) and then mapped to the genome using Bowtie2 v2.3.0 (Langmead and Salzberg 2012).

The contiguity and completeness of the *S. fusiforme* genome were assessed in comparison with the previously published assembly of *S. fusiforme* Ch. (Wang et al. 2020). Both of these assemblies were aligned using minimap2 v2.26 (Li 2018) and visualized using D-GENIES v1.5.0 (Cabanettes and Klopp 2018).

### Repeat Sequence Annotation

Before gene prediction, we identified genome-wide TE profiles of two *Sargassum* species using the de novo repeat library construction. To build the library, repeat elements from not only two *Sargassum* species genomes but also previously published brown algal genomes were identified using RepeatModeler v2.0.3 (Flynn et al. 2020) with -LTRStruct option. However, the results from RepeatModeler still contained numerous unclassified repeats because the database was designed for model organisms rather than algal lineages. To address this issue, reclassification of unclassified repeats was carried out using DeepTE (Yan et al. 2020). Subsequently, both *Sargassum* species genomes were masked based on the newly constructed repeat library using RepeatMasker v4.1.2 (Smit et al. 2013-2015). Kimura-distance of each TE in genomes from the results obtained from RepeatMasker was used for checking TE divergence.

### Gene Prediction and Functional Annotation

For gene prediction, gene expression data from both *Sargassum* species were produced by placing tissues under the four different conditions: light at 10 °C, dark at 10 °C, cold at 4 °C, and room temperature at 24 °C. The harvested samples were frozen using liquid nitrogen. RNA extraction was performed using the CTAB method, followed by treatment with DNase I. mRNA libraries were prepared using TruSeq Stranded mRNA kit and sequenced using NovaSeq6000 (Illumina, San Diego, USA). The transcriptome sequences were trimmed using Trimmomatic v0.39 (Bolger et al. 2014). They were aligned to the final sargassum genomes using STAR v2.7.10a with default parameters (Dobin et al. 2013). The transcript alignment information was converted into BAM files using Samtools v1.7 (Li et al. 2009).

Protein-coding genes of *S. thunbergii* and *S. fusiforme* were predicted using EVidenceModeler v.1.1.1 (Haas et al. 2008), which integrates ab initio gene prediction, protein homology-based prediction, and transcriptome-based prediction. Ab initio prediction was carried out using BRAKER v2.1.5 (Stanke et al. 2006; Stanke et al. 2008; Brůna et al. 2021) based on BAM files including transcript alignment information and soft-masked genomes. For protein homology-based prediction based on protein alignments, GeMoMa v.1.7.1 was employed (Keilwagen et al. 2019). To profile transcriptome-based gene models, the alignment assembly pipeline of PASA v2.0.2 was used (Haas et al. 2003), relying on de novo transcriptome assembly that referred to the assembled genomes via Trinity with the –genome_guided_bam option (Grabherr et al. 2011). Finally, a consensus gene model was constructed using EvidenceModeler with several rounds of weight adjustment.

BUSCO v5.7.1 with stramenopile odb10 and eukaryote odb10 datasets was utilized to assess the completeness of the genomes and gene models (Manni et al. 2021). Functional annotation of predicted genes was conducted with several methods. The results of BlastP (Camacho et al. 2009) against NCBI nr database (2023 February 20) and UniProtKB/Swiss-Prot database (UniProt Consortium 2023) with an *e*-value cutoff of 1e−05, eggNOG-Mapper v2.1.3 (Cantalapiedra et al. 2021) with the eggNOG database (Huerta-Cepas et al. 2019), InterProScan v5.52-86.0 (Jones et al. 2014), and KEGG Automatic Annotation Server (KAAS) (Moriya et al. 2007) were compared.

### Identification of Duplication Genes and *K*_a_/*K*_s_ Analysis

Duplication genes and duplication modes were identified following *DupGen_finder* pipeline (Qiao et al. 2019). Protein sequences of brown algal genomes were compared using local BlastP (1e−10 e-value, top 5 matches, and output format 6). *Tribonema minus* was selected as the outgroup (Mahan et al. 2021). *K*_a_, *K*_s_, and *K*_a_/*K*_s_ of duplicated pairs were calculated following *calculate_Ka_Ks_pipeline* based on KaKs_Calculator v2.0 (Wang et al. 2010; Qiao et al. 2019).

### Orthologue Analysis and Comparison of Gene Inventory

Orthologous gene families (OGFs) were clustered using OrthoFinder v2.5.2 with -M msa option (Emms and Kelly 2019). Protein sequences from two *Sargassum* genomes were compared with published gene models of seven brown algae, six stramenopiles, two oomycetes, four plant lineages, and three red algae (described information in supplementary fig. S9, Supplementary Material online) for the OGF analysis.

The gain and loss of genes were estimated using Count with the Dollo parsimony principle (Csűös 2010). Gain and loss analysis was conducted using “*extract_dollop_output_sequences_v2-fast.pl*” script (github.com/guyleonard/orthomcl_tools). Highly duplicated genes in *Sargassum* genomes were identified using three criteria: (i) they should not be of foreign origin, (ii) the average gene count in *Sargassum* should be twice as high as in other brown algae, and (iii) the minimum gene count among the three *Sargassum* species should be at least 1.5 times higher than the maximum gene count in five other brown algal species.

The COG category was investigated to identify functional differences of duplicated, gained, and lost genes based on the result from eggNOG-Mapper v2.1.3 (Cantalapiedra et al. 2021) with the eggNOG database (Huerta-Cepas et al. 2019). Gene content of the three *Sargassum* genomes and their putative functions were compared using Orthovenn3 (Sun et al. 2023).

### Experimental Design for Desiccation Stress Reaction and Transcriptome Sequencing

To profile transcriptional reaction against abiotic stress, we selected and tested desiccation stress, which is commonly faced in natural habitats for the *Sargassum* species. Clean apical tips of *S. fusiforme* and *S. thunbergii* thallus were cut into ∼1-cm lengths and precultured in sterile seawater under controlled culture conditions (12-h light/12-h dark photoperiod at 15 °C) for 3 d before beginning the experiment. Nonstressed control samples were continually supplied with sterile seawater, while other samples were subjected to desiccation stress by removing seawater and exposing to the ambient atmosphere in the chamber (15 °C) for 6 h. For each experimental setting, three fully independent data sets (three apical parts per container) were generated. All samples were stored at −80 °C until used for RNA extraction. Total RNAs were extracted from three independent apical parts using a CTAB-based extraction. For mRNA sequencing, the TruSeq Stranded Total RNA with Ribo-Zero Plant library kit was used, and amplified libraries were paired-end sequenced (2 × 100 bp) with Illumina NovaSeq6000 platform (supplementary table S17, Supplementary Material online). Additionally, the total RNAs of *S. fusiforme* and *S. thunbergii* were used for cDNA synthesis to compare gene expression of the SA pathway using further quantitative real-time PCR (qRT-PCR) experiments.

### DEGs and GO Enrichment Analysis

The RNA-seq reads were quality-trimmed by using Trimmomatic v0.39 (Bolger et al. 2014), and trimmed reads were aligned to *S. fusiforme* and *S. thunbergii* draft genomes using STAR v2.7.10a with default parameters (Dobin et al. 2013). Gene expression levels were determined using Cufflinks with calculations based on fragments per kilobase of exon per million mapped fragments values (Trapnell et al. 2010). DEG analysis was performed using Cuffdiff, following the utilization of Cuffmerge to merge data from four different conditions (Trapnell et al. 2012).

DEGs for downstream analysis were considered significant if they met the criteria of having a false discovery rate that was significant (<0.05) after Benjamini–Hochberg correction. DEGs were identified by considering genes with a log_2_FC (fold change) ≤−2 or ≥2 for *S. fusiforme* and a log_2_FC ≤−1.2 or ≥1.2 for *S. thunbergii*. Moreover, as DEGs were occasionally assigned based on the presence of outlier values in a single gene, *Z*-score was employed to exclude genes displaying values <−2 or >2.

Principal component analysis (supplementary fig. S14, Supplementary Material online) and correlation of replicated datasets (supplementary fig. S15, Supplementary Material online) were carried out using the psych R package (Revelle 2023). GO enrichment analysis was performed using DAVID (Sherman et al. 2022). Since our genomes were de novo assembled and there were no *Sargassum*-specific databases to identify GOs, all DEGs were matched with the UniProtKB entries of the model brown algae, *E. siliculosus* (https://www.uniprot.org/taxonomy/2880) (Cock et al. 2010), based on the blastp result against UniProtKB/Swiss-Prot database (The UniProt Consortium 2023). The functional annotation of DEGs was mainly adopted from the Pfam domain information within the InterProScan annotation results.

### Discovery of Genes Related to SA

To classify detailed genes involved in the SA pathway, we adopted the representative pathway robustly studied in a model plant (*Arabidopsis thaliana*) (Yang et al. 2015). Due to the evolutionary distance between the plant and brown algal lineages, we searched for genes based on the composition of main domains rather than sequence similarity search. To this end, the domain information was retrieved from UniProtKB/Swiss-Prot. We searched SA pathway genes satisfying to contain all retrieved main domains based on InterProScan results (supplementary table S9, Supplementary Material online). Furthermore, we equally searched the genes within gene models from unmasked genomes of both species to explore any missing genes from the neglected genomic regions. Gene expression levels were calculated using the same method as the above RNA-seq analysis.

### qRT-PCR Validation

To explore the gene expression of the SA synthesis pathway under desiccation stress, qRT-PCR was conducted. cDNA was prepared from 2 µg of each DNase-treated RNA, using the RevertAid First Strand cDNA Synthesis Kit according to the manufacturer's instructions (Fermentas, Lithuania), and subsequently used in a quantitative PCR (qPCR) reaction. qPCRs were performed using SsoFast EvaGreen Supermix (Bio-Rad, Hercules, CA, USA) on a CFX connect Real-Time System (Bio-Rad, Hercules, CA, USA) with Bio-Rad CFX Manager software (Bio-Rad, Hercules, CA, USA). Primers were designed using the Primer 3 (Rozen and Skaletsky 1999) website, and their sequences are given in in supplementary table S11, Supplementary Material online. Preliminary quantitative PCR assay and melting curve analysis were performed to evaluate efficiency of primer pairs and absence of genomic DNA contamination and to ensure amplification of a single product. As reference genes for standardization, we used an EF1*β* gene and an EF2*α* gene, which we had newly identified as suitable housekeeping genes with the least variable expression. All reactions were run in three biological replicates. The relative quantification of each gene expression among samples was evaluated by a comparative ΔΔC_T_ method.

### Subcellular Localization Analysis

Two DNA fragments encoding two putative EDS1 isoforms were amplified by PCR with their specific primer pairs using cDNA as a template. The primer sequences are described in supplementary table S12, Supplementary Material online. The PCR products were digested with *Xba*I and *Bam*HI and ligated in-frame upstream of the GFP gene of the vector 326-sGFP. All constructs were validated by sequencing. To analyze the subcellular localization, *Arabidopsis* leaf protoplasts were isolated according to Yoo et al. (2007). The in-frame GFP fusion constructs were introduced into *Arabidopsis* protoplasts by the PEG-mediated method (Abel and Theologis 1994) and then incubated for 18 to 60 h in the dark. Images of GFP and red chlorophyll autofluorescence in the transformed protoplasts were acquired by a cooled CCD camera and an Olympus BX53 Light/Fluorescence microscope at 40× magnification.

### Extraction and Sample Preparation for SA Analysis

For the quantitative analysis of SA in *Sargassum* species, we compared the SA content between control groups and desiccation stress treatment groups (6 and 12 h) for both species. All samples were weighed and then freeze-dried (supplementary table S9, Supplementary Material online). The freeze-dried samples were ground into powder and ultrasonically extracted twice at 55 °C for 1 h each using 10 times the sample weight of methanol (MeOH). Each MeOH extract 100 mg was precisely weighed, 2 mL of hexane was added, sonicated at 50 °C for 10 min, and centrifuged at 3,000 rpm for 10 min, and then, the hexane layer was removed. This process was repeated twice. Ethyl acetate (EtOAc) 2 mL was added to the dried residue and ultrasonic extraction was performed twice at 50 °C for 10 min. The EtOAc layers obtained by centrifugation at 3,000 rpm for 10 min were dried under nitrogen streams. The EtOAc extracts (100 mg) were dissolved in acetonitrile (MeCN) 500 μL by sonication at 50 °C for 10 min and then centrifuged at 3000 rpm for 10 min. The supernatants were used as samples for quantitative analysis of SA.

### Preparation of Standard

The SA and ibuprofen (IS) were dissolved in MeCN as concentrations of 1,000 and 4,000 μg/mL, respectively. The standard working solutions of SA were prepared by serial dilution with MeCN, to give 200, 100, 50, 25, 12.5, and 6.25 ng/mL. The IS working solution was prepared by dilution with MeCN, yielding a concentration of 2,000 ng/mL. The sample and standard solutions were spiked with the IS working solution to a final IS concentration of 100 ng/mL.

### HR-MS and UHPLC–MS/MS Analysis

HR-MS data for SA was obtained from an Ultimate 3000/Q Exactive UHPLC/Q Oribitrap High Resolution Mass Spectrometer (Thermo Scientific, USA) using negative ion mode.

UHPLC–MS/MS analysis was performed using an ExionLC (AB SCIEX, USA) coupled with a Triple Quad 6500 (AB SCIEX, USA). The analysis was carried out using an ACQUITY UPLC HSS C18 100 Å 1.8 µm column (2.1 × 150 mm, Waters Corporation, USA). The eluent consisted of water (A) and 0.1% formic acid in MeCN (B). The gradient profile was as follows: 0 to 3.5 min, 50% A; 3.5 to 6 min, 50% to 5% A; 6 to 9 min, 5% A; 9 to 9.1 min, 5% to 50% A; and 9.1 to 12 min, 50% A. The flow rate, column oven temperature, and injection volume were set at 0.2 mL/min, 30 °C, and 5 μL, respectively.

The UHPLC system was coupled to a Triple Quad 6500 triple quadrupole mass spectrometer equipped with an electrospray ionization source. The instrumental conditions including curtain gas, collision gas, ion spray voltage, temperature, ion source gas 1, and ion source gas 2 were set at 20 psi, 8 psi, −4,500 V, 330 °C, 40 psi , and 40 psi, respectively. The instrument was operated in a negative ion mode, and the analytes were detected using MRM mode. In the MRM mode, the transitions of precursor ion to product ion were monitored at *m/z* 136.9 → 92.9 in the quantification and *m/z* 136.9 → 65.1 for the qualification of SA, while *m/z* 205 → 159 in the quantification and *m/z* 205 → 161 in the qualification for ibuprofen as the IS.

## Supplementary Material

evaf084_Supplementary_Data

## Data Availability

*Sargassum fusiforme* and *S. thunbergii* genome assemblies and gene models were uploaded to the figshare under the doi link: https://doi.org/10.6084/m9.figshare.27682986. Raw data used for genome assemblies were deposited on NCBI under BioProjects (PRJNA1185288 and PRJNA1184803). Raw transcriptomic data for desiccation stress experiments were also deposited on NCBI under PRJNA1184453.
