# Supplementary material for: Genome Evolution of Two Intertidal Sargassum Species (S. fusiforme and S. thunbergii) and Their Response to Abiotic Stressors
Source: Genome Biol Evol. 2025 May 3;17(5):evaf084. doi: 10.1093/gbe/evaf084 (PMC12089773; doi:10.1093/gbe/evaf084)
Supplement: evaf084_Supplementary_Data [file evaf084_supplementary_data.zip › Revised_Supplementary legends_250430.docx]

**Table S1. Summary of produced PacBio reads from the** *S. thunbergii* **and *S.*** *fusiforme* **assemblies.**

**Table S2.** Summary of TEs of the *S. thunbergii,* ***S.*** *fusiforme*, and kelp genomes.

**Table S3.** Summary of genome features and BUSCOs of the *S. thunbergii* **and *S.*** *fusiforme* genomes.

**Table S4.** Results of GO enrichment analysis based on orthologous cluster analysis of Sargassum genomes using OrthoVenn3.

**Table S5.** Number of duplicated genes in Sargassum and brown algal genome detected by *DupGen_finder* pipeline.

**Table S6.** Ka/Ks, Ka, and Ks of duplicated genes in *Sargassum* and brown algal genome.

**Table S7.** Summary of main duplicated genes of *S. thunbergii* and ***S.*** *fusiforme* genomes compared to kelp genomes.

**Table S8.** Assigned Orthologous Groups (OGs) in *Sargassum* using OrthoFinder v2.5.2 and Count with the Dollo parsimony principle analyses.

**Table S9.** Summary of DEGs of *S. thunbergii* and ***S.*** *fusiforme* genomes under desiccation stress.

**Table S10.** Domain information and identification results of selected salicylic acid pathway genes in the *Sargassum* genomes.

**Table S11.** Primer information for qRT-PCR for salicylic acid synthesis pathway genes of *S. thunbergii* **and *S.*** *fusiforme* genomes against desiccation stress.

**Table S12.** Primer information for putative subcellular localization of *EDS1*.

**Table S13.** Weight of used *Sargassum* samples and amount of the methanol (MeOH) extracts.

**Table S14.** UHPLC-MS/MS results of control and desiccation treatment groups of *Sargassum.*

**Table S15.** Summary of Fucales genomes.

**Table S16.** Summary of Illumina reads for the *S. thunbergii* **and *S.*** *fusiforme* assemblies.

**Table S17.** Summary of produced Illumina reads for DEG analyses.

**Figure S1.** Detection and elimination of contaminants in *S. thunbergii* **and *S.*** *fusiforme* data using BlobTools v1.1.

**Figure S2.** Genome size estimation and its failure using short-read data from *S. thunbergii* and *S. fusiforme*.

**Figure S3.** Improved continuity and comparison of the newly assembled *S. fusiforme* genome from the Korean population with the *S. fusiforme* genome from the Chinese population.

**Figure S4.** Frequency and distribution of Kimura distance of TEs of *S. thunbergii* **and *S.*** *fusiforme*.

**Figure S5.** Functional annotation of the *S. thunbergii* genome after gene prediction based on KAAS, Uniport, Eggnog, Interproscan, and the nr NCBI database.

**Figure S6.** Orthologous cluster analysis of the newly assembled *S. thunbergii* and *S. fusiforme*, and previously published *S. fusiforme* Ch using OrthoVenn3.

**Figure S7.** Ka and Ks value of duplicated genes in different modes in *Sargassum* and brown algal genomes. The Kruskal-Wallis H test was conducted to compare Ka and Ks values across species. All comparisons were significant differenent (*p*<0.01). Post hoc pairwise comparisons were carried out using Dunn's test, with distinct alphabet letters indicating statistically significant differences between groups. a) Ka values. b) Ks values.

**Figure S8.** Clusters of Orthologous Groups (COGs) category of gene gain and loss analysis. The number of OGs in each clade was designated using OrthoFinder v2.5.2 and Count with the Dollo parsimony principle, as shown in Figure S9. Detailed information on the assigned OGs is provided in Supplementary Table S8.

**Figure S9.** Results of dollo parsimony and gene gain and loss analysis based on orthologue comparisons. *S. thunbergii*, *S. fusiforme*, six brown algae species, stramenopiles (*Aureococcus anophagefferens*; Gobler et al. 2011, *Fragilariopsis cylindrus*; Mock et al. 2017, *Phaeodactylum tricornutum*; Bowler et al. 2008, *Thalassiosira pseudonana*; Armbrust et al. 2004, *Nannochloropsis gaditana*; Radakovits et al. 2011, *Tribonema minus*; Mahan et al. 2021), oomycetes (*Thraustotheca clavata*; Misner et al. 2014, *Peronospora effusa*; Fletcher et al. 2022), plant lineages (*Arabidopsis thaliana*; The Arabidopsis Genome Initiative, 2000, *Chlamydomonas reinhardtii*; Merchant et al. 2007, *Oryza sativa*; Kawahara et al. 2013, *Ostreococcus tauri*; Derelle et al. 2006), and red algae (*Cyanidioschyzon merolae*; Matsuzaki et al. 2004, *Chondrus crispus*; Collén et al. 2013, *Gracilariopsis chorda*; Lee et al. 2018) were compared.

**Figure S10.** Clusters of Orthologous Groups (COGs) category of gene gain and loss analysis. The number of OGs in each clade was designated using OrthoFinder v2.5.2 and Count with the Dollo parsimony principle, as shown in Figure S9. Detailed information on the assigned OGs is provided in Supplementary Table S8.

**Figure S11.** Method for extraction of salicylic acid from *Sargassum* samples.

**Figure S12.** Calibration curve, regression equation, and correlation coefficient (R') for the quantification of salicylic acid.

**Figure S13.** MRM chromatograms of salicylic acid at *m/z* 136.9 → 92.9 in *Sargassum* samples for the control and desiccation stress groups (6 and 12 h)*.* Triple quadrupole mass spectrometer was operated in a negative ion mode and the separated analytes were detected using MRM mode at *m/z* 136.9 → 92.9 for the quantification and *m/z* 136.9 → 65.1 for the qualification of salicylic acid. Ibuprofen was used as an internal standard.

**Figure S14.** PCA of transcriptome datasets of *S. thunbergii* and *S*. *fusiforme* against desiccation stress.

**Figure S15.** Correlation of transcriptome datasets of *S. thunbergii* **and *S.*** *fusiforme* against desiccation stress.
