# Supplementary material for: Genome Evolution of Two Intertidal Sargassum Species (S. fusiforme and S. thunbergii) and Their Response to Abiotic Stressors
Source: Genome Biol Evol. 2025 May 3;17(5):evaf084. doi: 10.1093/gbe/evaf084 (PMC12089773; doi:10.1093/gbe/evaf084)
Supplement: evaf084_Supplementary_Data [file evaf084_supplementary_data.zip › Revised_Supplementary_figures_v7_GBE_250430.docx]

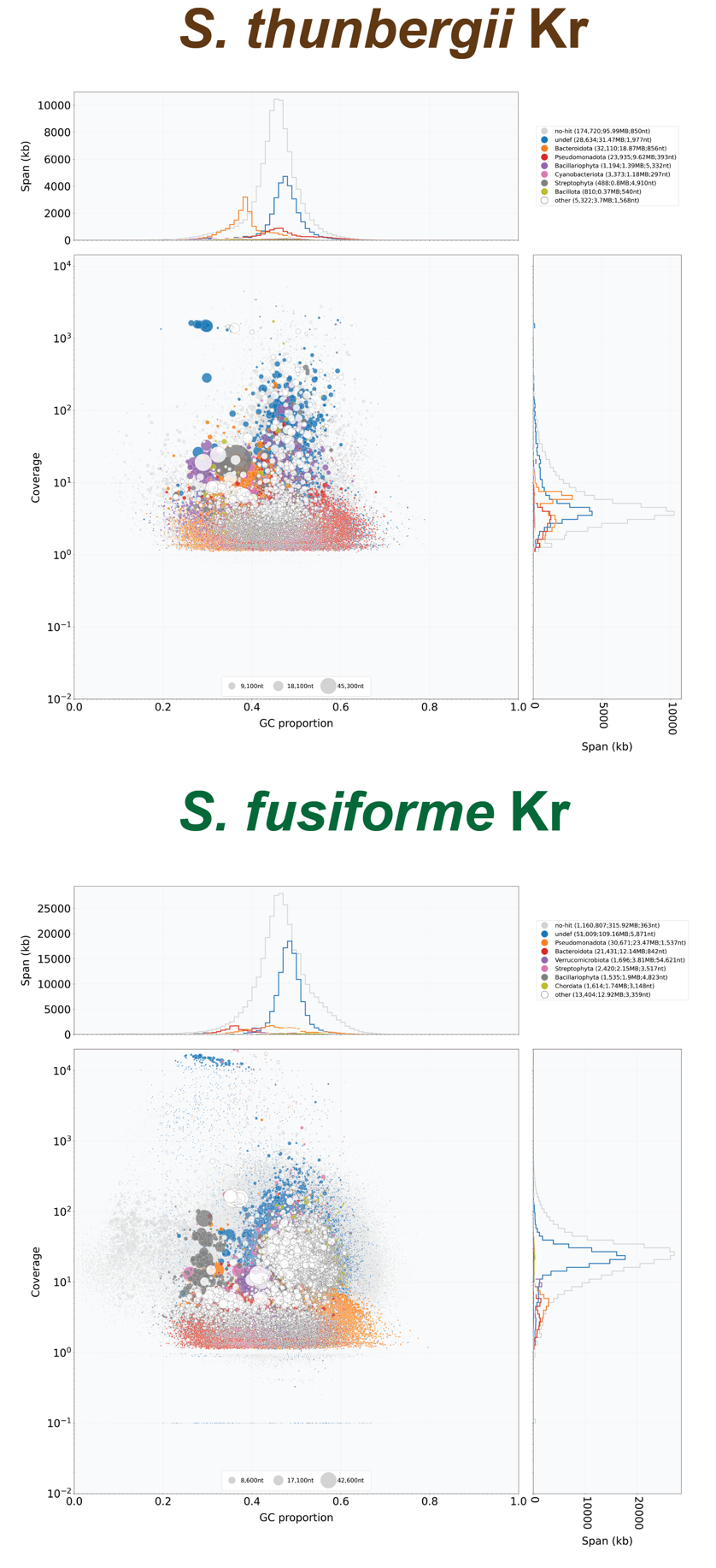


**Figure S1.** Detection and elimination of contaminants in *S. thunbergii* **and *S.*** *fusiforme* data using BlobTools v1.1.


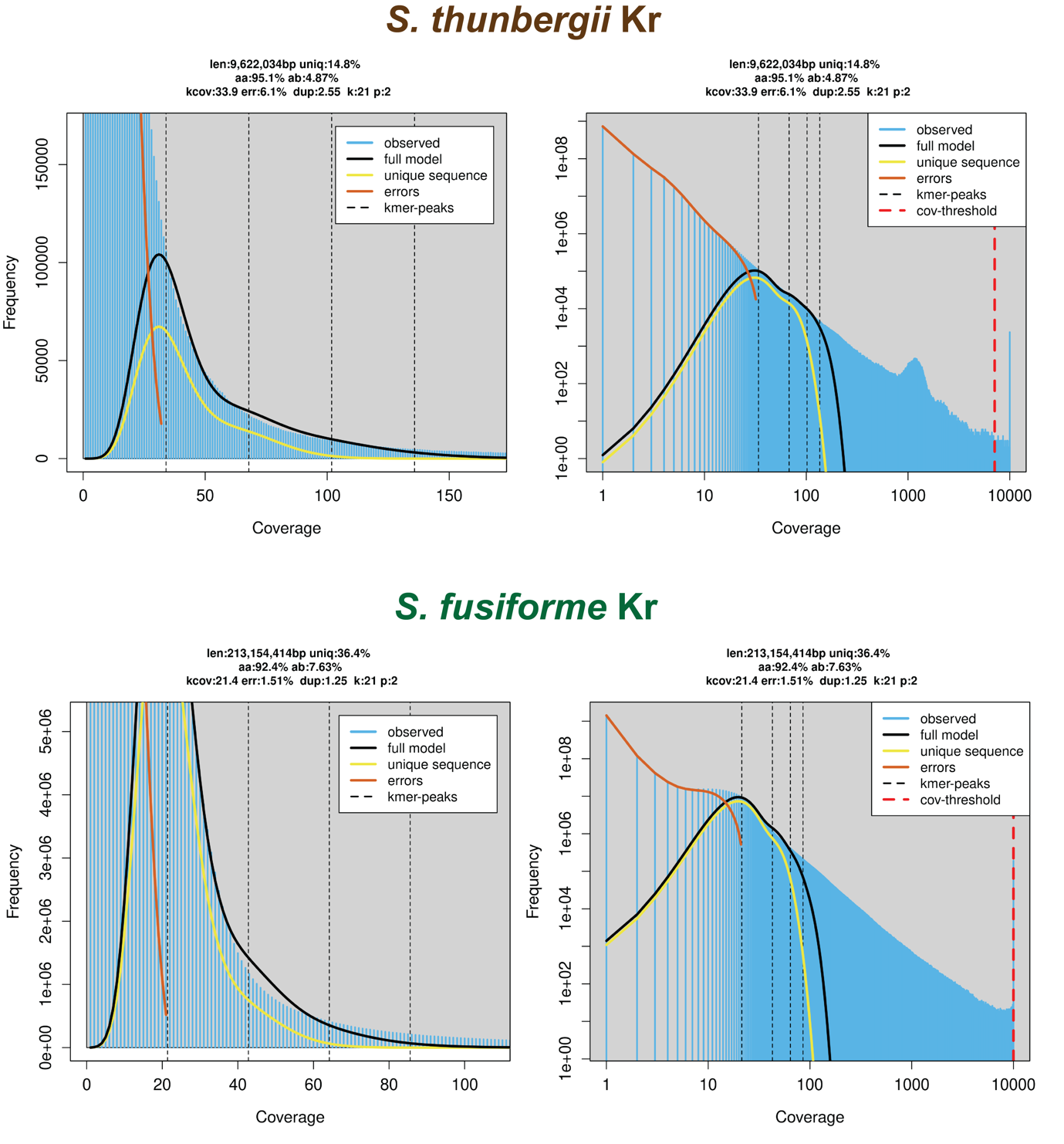


**Figure S2.** Genome size estimation and its failure using short-read data from *S. thunbergii* and *S. fusiforme*.


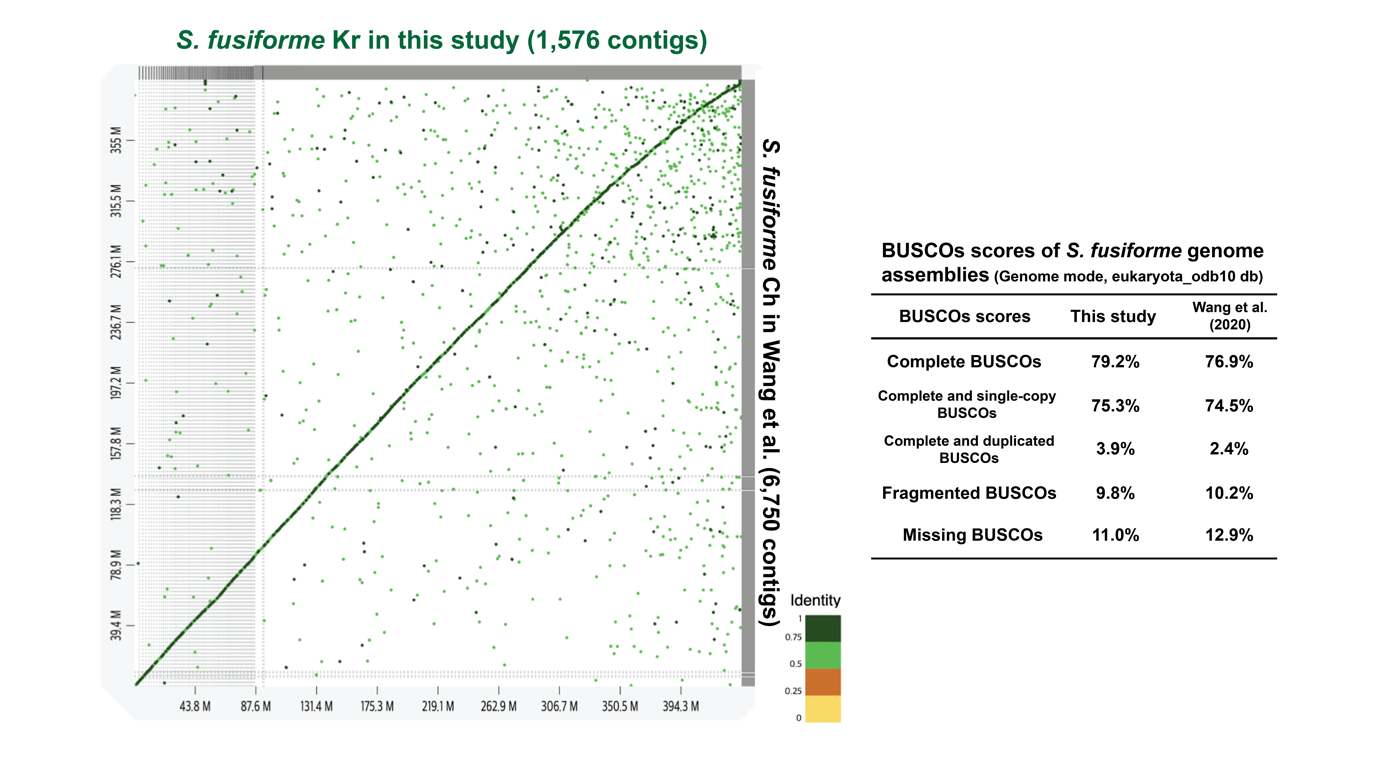


**Figure S3.** Improved continuity and comparison of the newly assembled *S. fusiforme* genome from the Korean population with the *S. fusiforme* genome from the Chinese population.


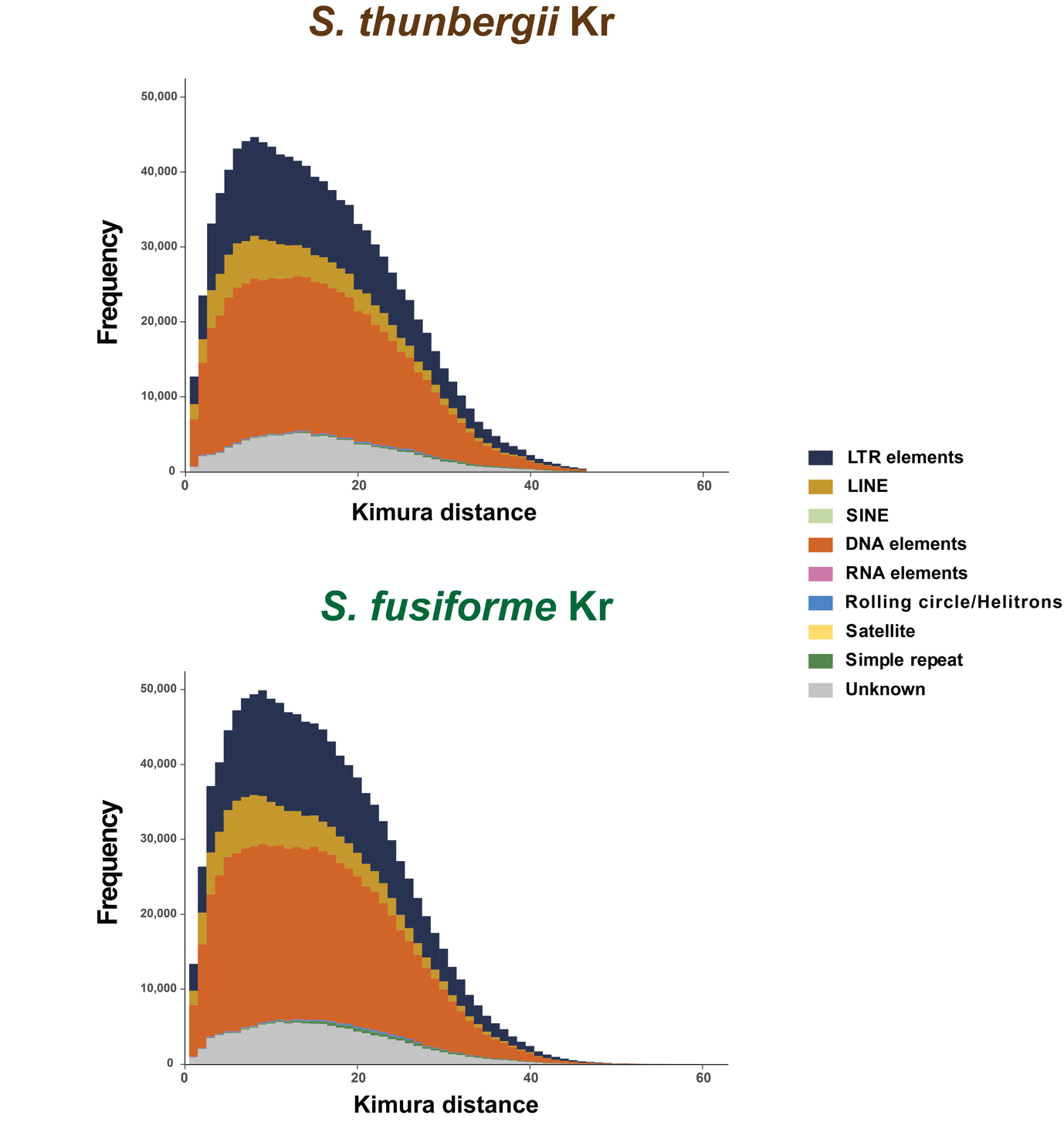


**Figure S4.** Frequency and distribution of Kimura distance of TEs of *S. thunbergii* **and *S.*** *fusiforme*.


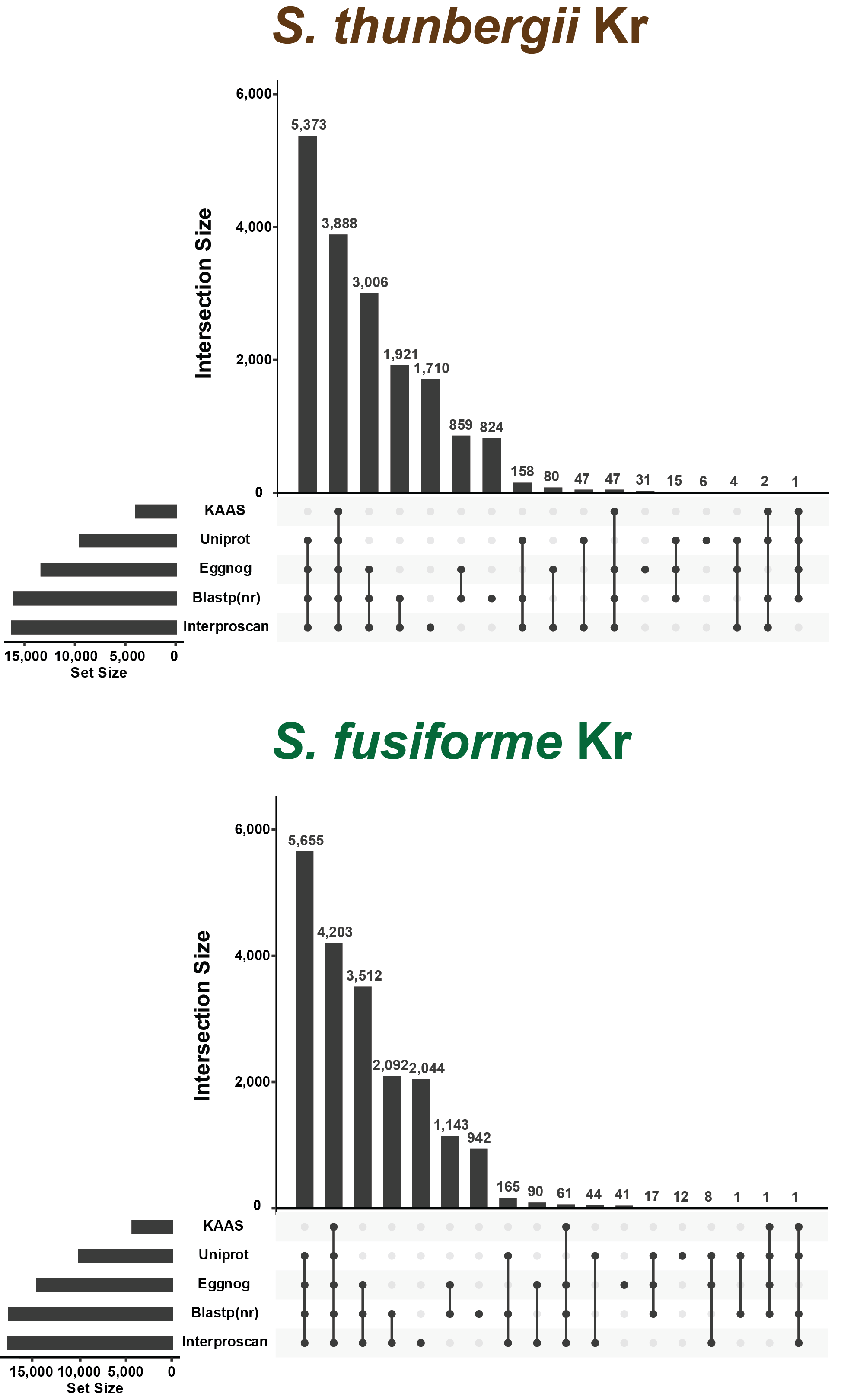


**Figure S5**. Functional annotation of the *S. thunbergii* genome after gene prediction based on KAAS, Uniport, Eggnog, Interproscan, and the nr NCBI database.


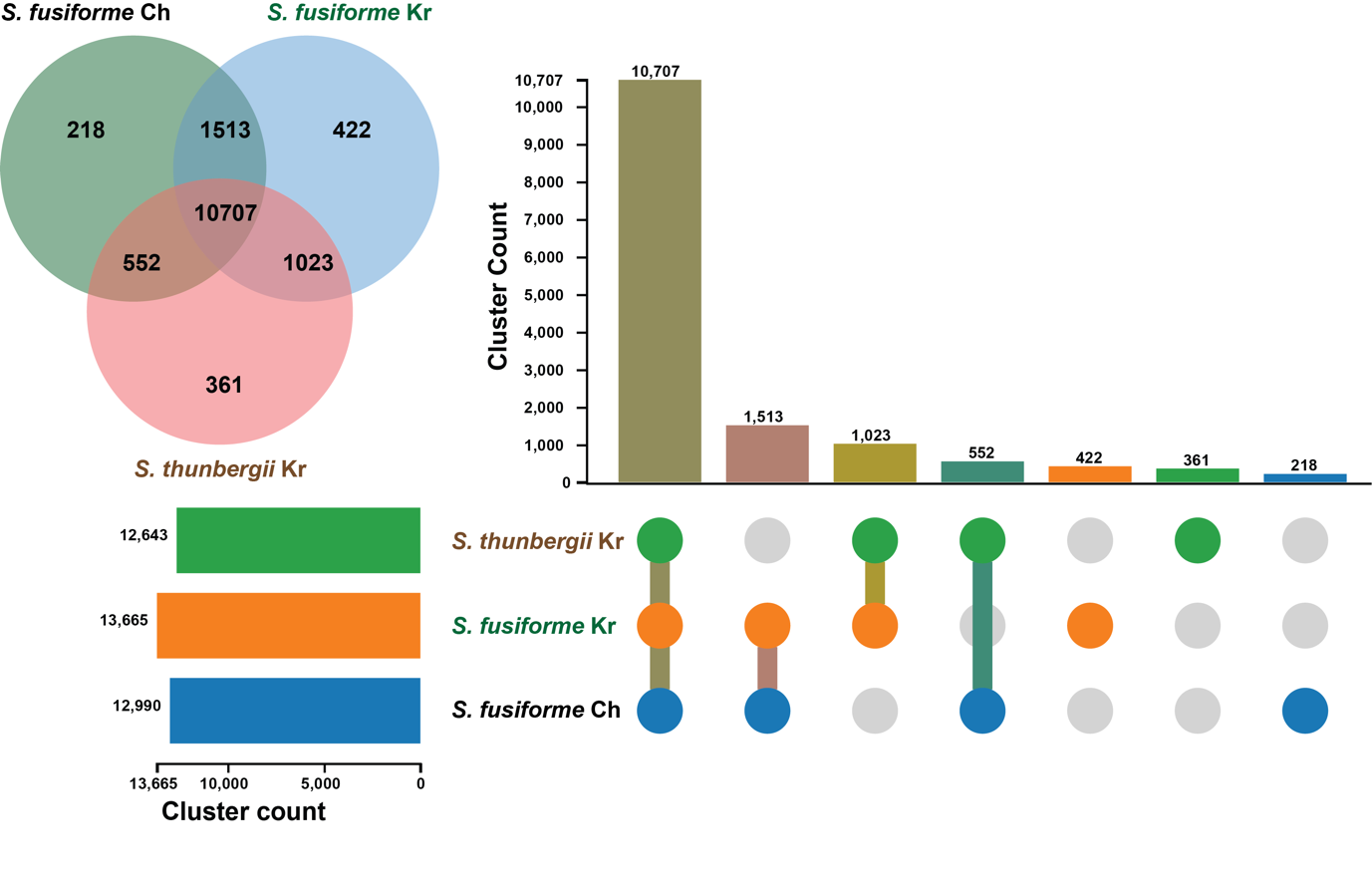


**Figure S6**. Orthologous cluster analysis of the newly assembled *S. thunbergii* and *S. fusiforme*, and previously published *S. fusiforme* Ch using OrthoVenn3.


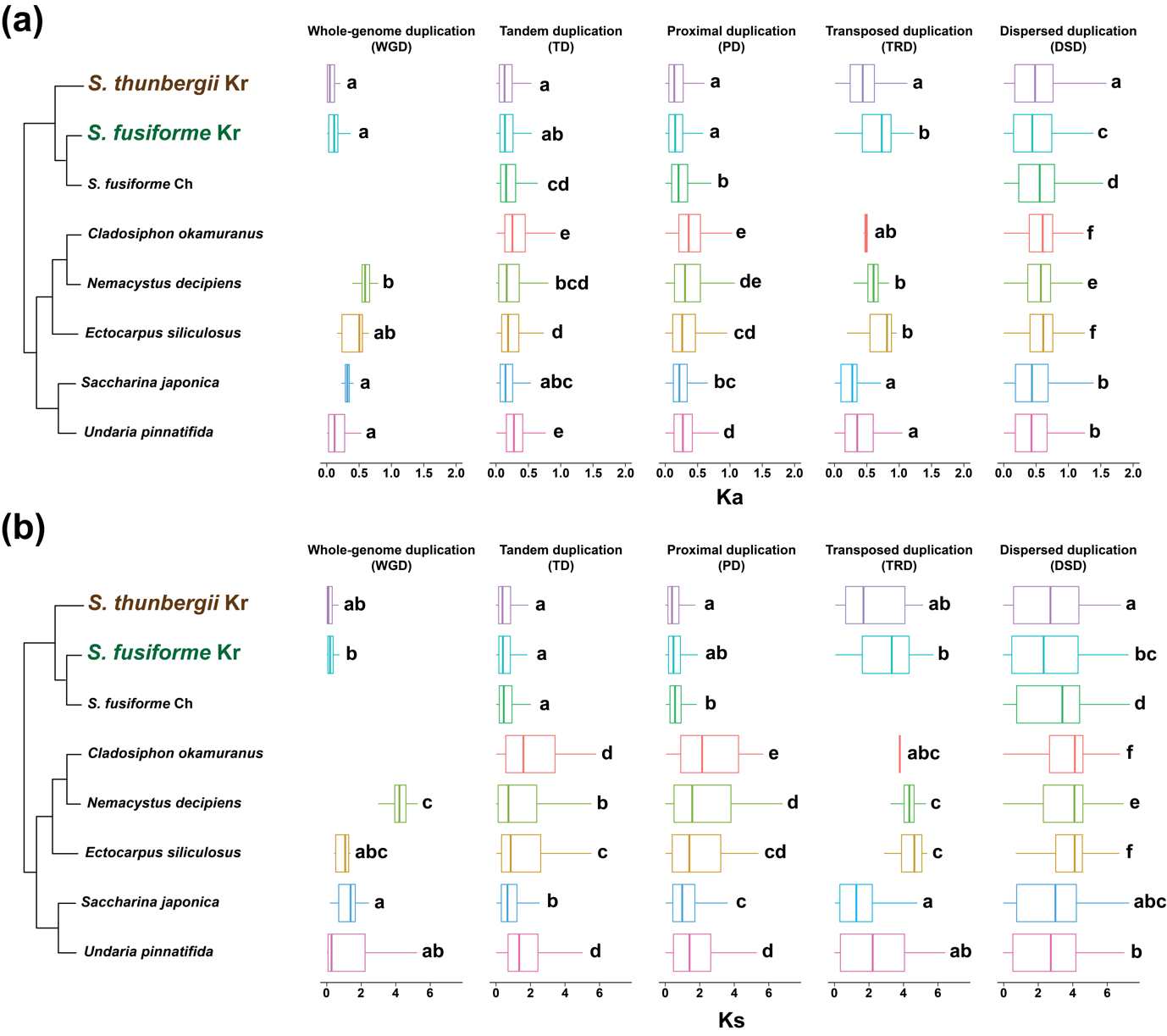


**Figure S7**. Ka and Ks value of duplicated genes in different modes in *Sargassum* and brown algal genomes. The Kruskal-Wallis H test was conducted to compare Ka and Ks values across species. All comparisons were significant differenent (*p*<0.01). Post hoc pairwise comparisons were carried out using Dunn's test, with distinct alphabet letters indicating statistically significant differences between groups. a) Ka values. b) Ks values.


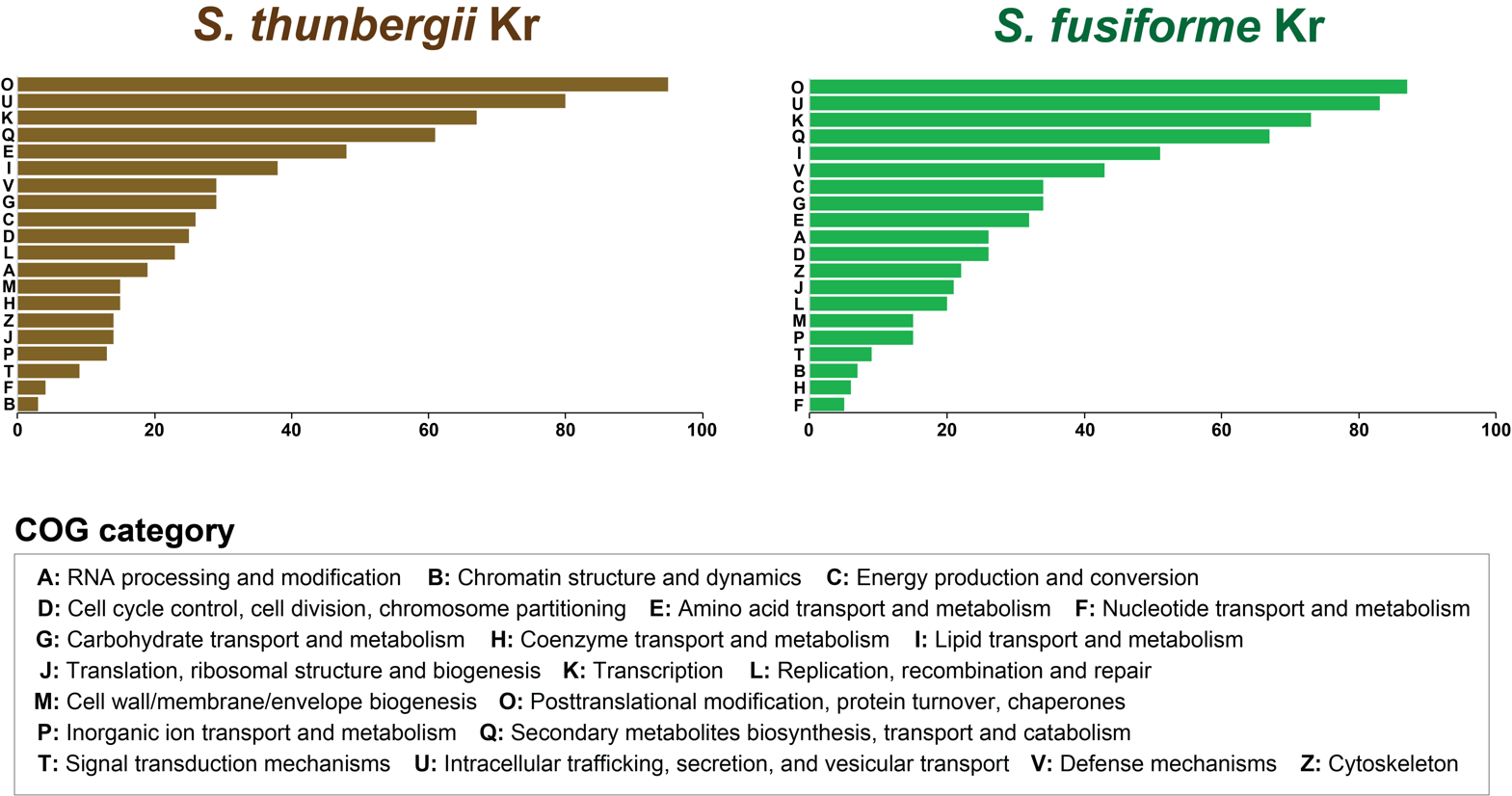


**Figure S8**. Clusters of Orthologous Groups (COGs) category of significant duplicated genes.


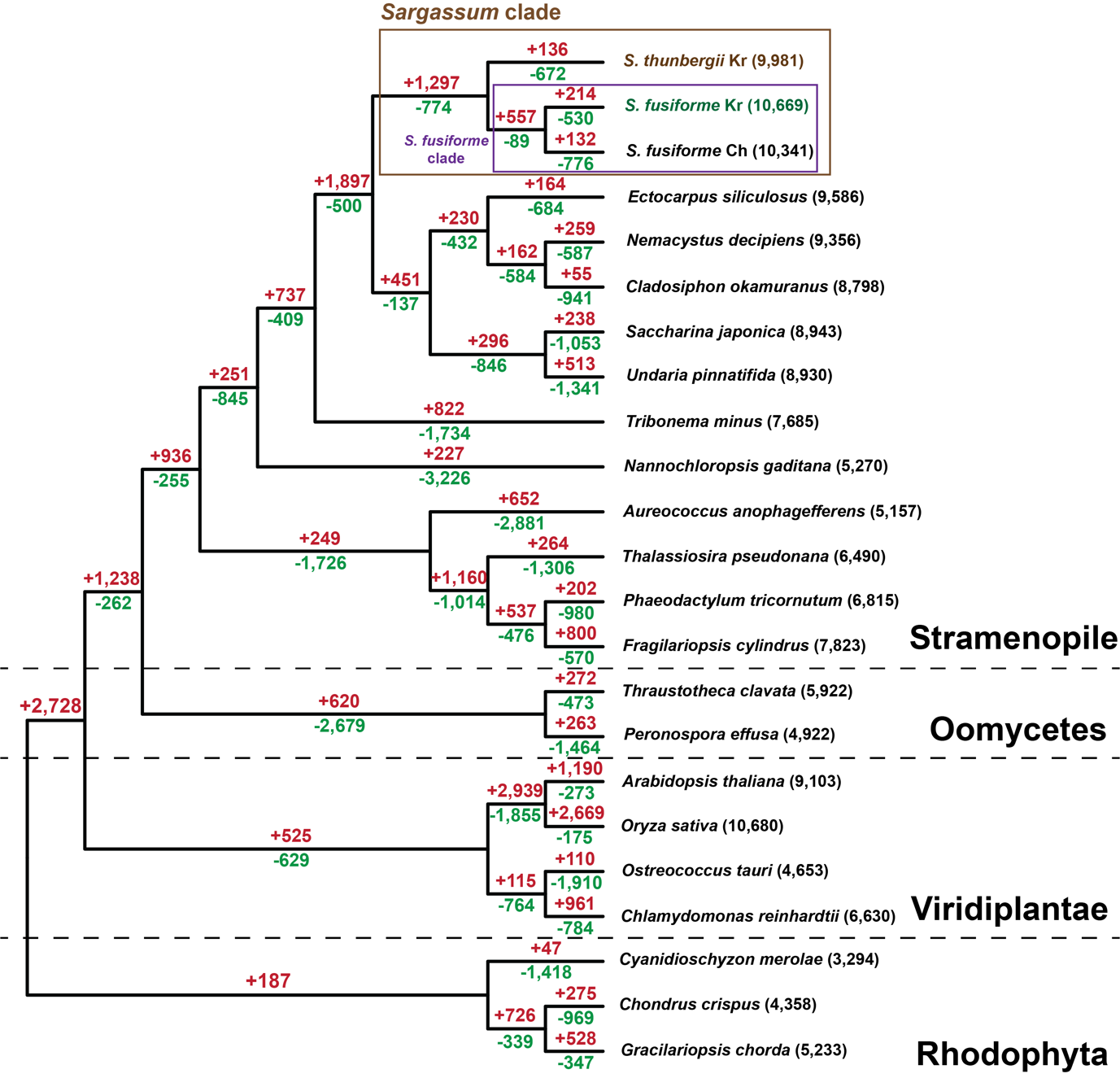


**Figure S9.** Results of dollo parsimony and gene gain and loss analysis based on orthologue comparisons. *S. thunbergii*, *S. fusiforme*, six brown algae species, stramenopiles (*Aureococcus anophagefferens*; Gobler et al. 2011, *Fragilariopsis cylindrus*; Mock et al. 2017, *Phaeodactylum tricornutum*; Bowler et al. 2008, *Thalassiosira pseudonana*; Armbrust et al. 2004, *Nannochloropsis gaditana*; Radakovits et al. 2011, *Tribonema minus*; Mahan et al. 2021), oomycetes (*Thraustotheca clavata*; Misner et al. 2014, *Peronospora effusa*; Fletcher et al. 2022), plant lineages (*Arabidopsis thaliana*; The Arabidopsis Genome Initiative, 2000, *Chlamydomonas reinhardtii*; Merchant et al. 2007, *Oryza sativa*; Kawahara et al. 2013, *Ostreococcus tauri*; Derelle et al. 2006), and red algae (*Cyanidioschyzon merolae*; Matsuzaki et al. 2004, *Chondrus crispus*; Collén et al. 2013, *Gracilariopsis chorda*; Lee et al. 2018) were compared.


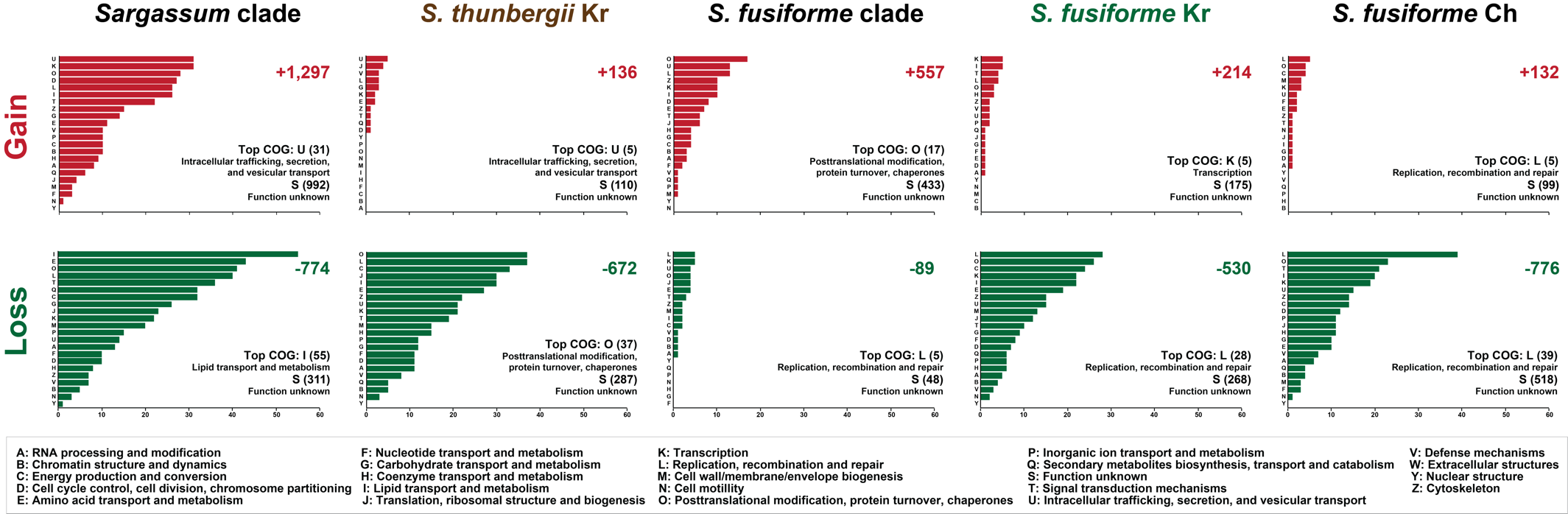


**Figure S10.** Clusters of Orthologous Groups (COGs) category of gene gain and loss analysis. The number of OGs in each clade was designated using OrthoFinder v2.5.2 and Count with the Dollo parsimony principle, as shown in Figure S9. Detailed information on the assigned OGs is provided in Supplementary Table S8.


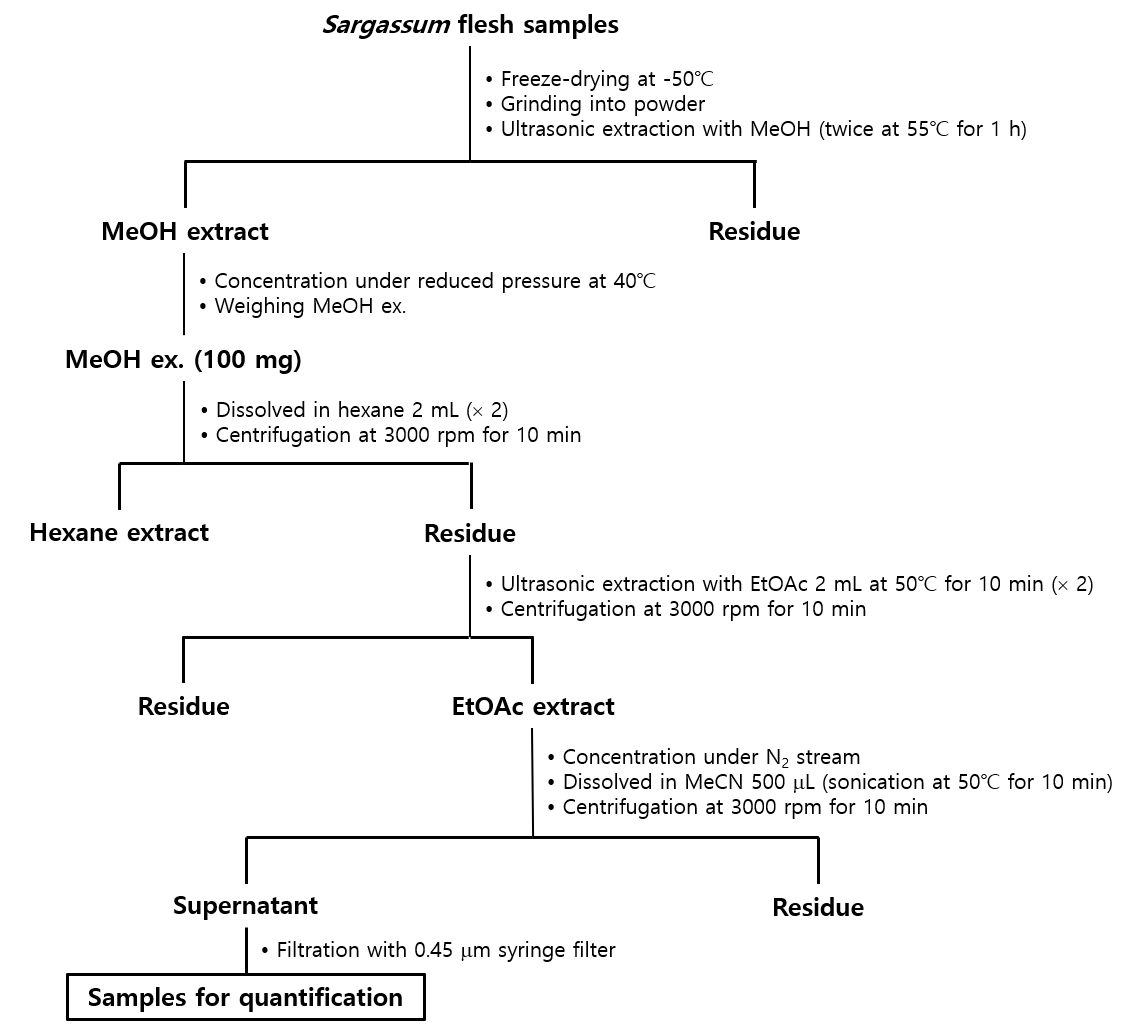


**Figure S11.** Method for extraction of salicylic acid from *Sargassum* samples.

**Figure S12.** Calibration curve, regression equation, and correlation coefficient (R') for the quantification of salicylic acid.


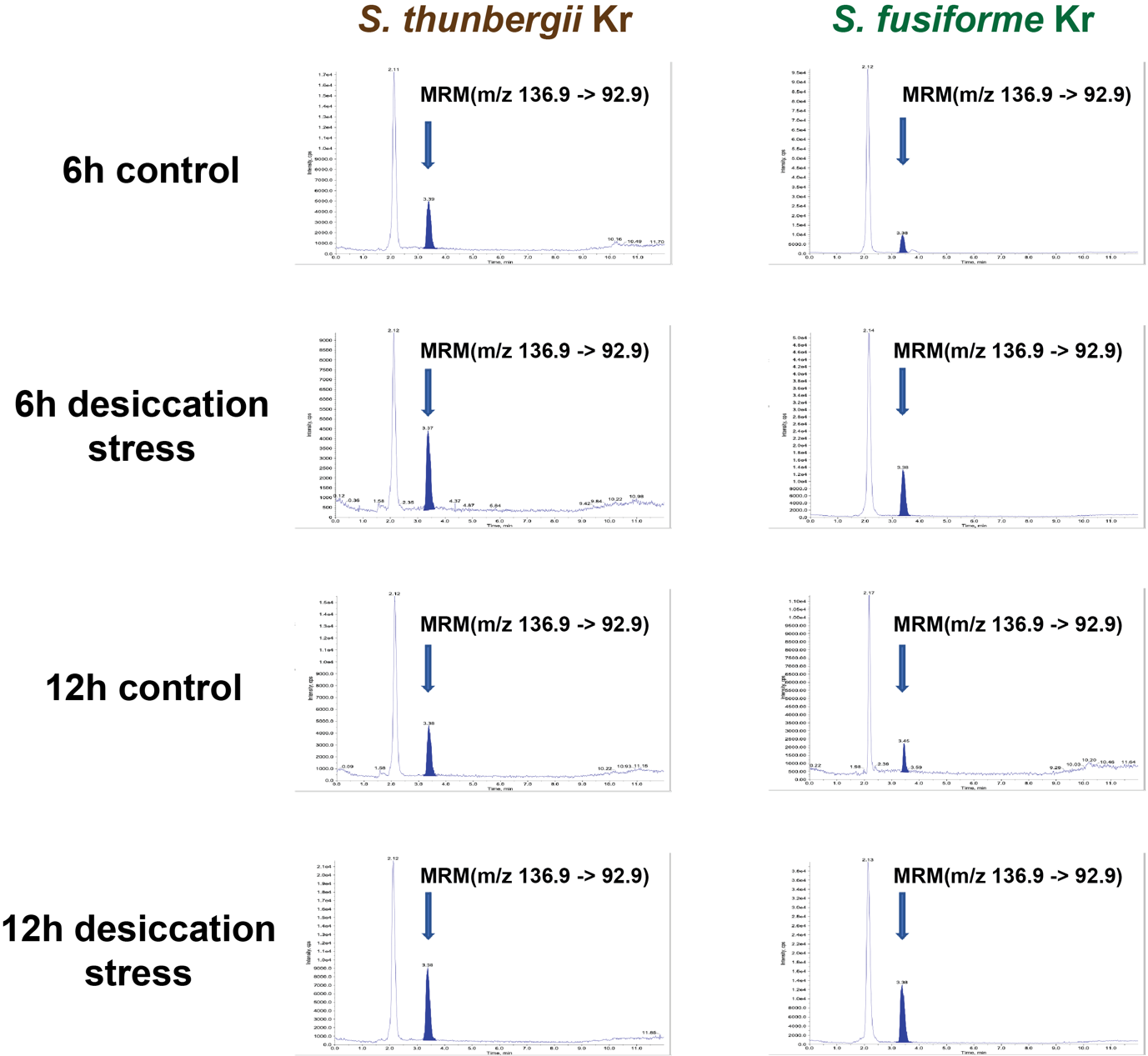


**Figure S13.** MRM chromatograms of salicylic acid at *m/z* 136.9 → 92.9 in *Sargassum* samples for the control and desiccation stress groups (6 and 12 h)*.* Triple quadrupole mass spectrometer was operated in a negative ion mode and the separated analytes were detected using MRM mode at *m/z* 136.9 → 92.9 for the quantification and *m/z* 136.9 → 65.1 for the qualification of salicylic acid. Ibuprofen was used as an internal standard.

**Figure S14.** PCA of transcriptome datasets of *S. thunbergii* and *S*. *fusiforme* against desiccation stress.


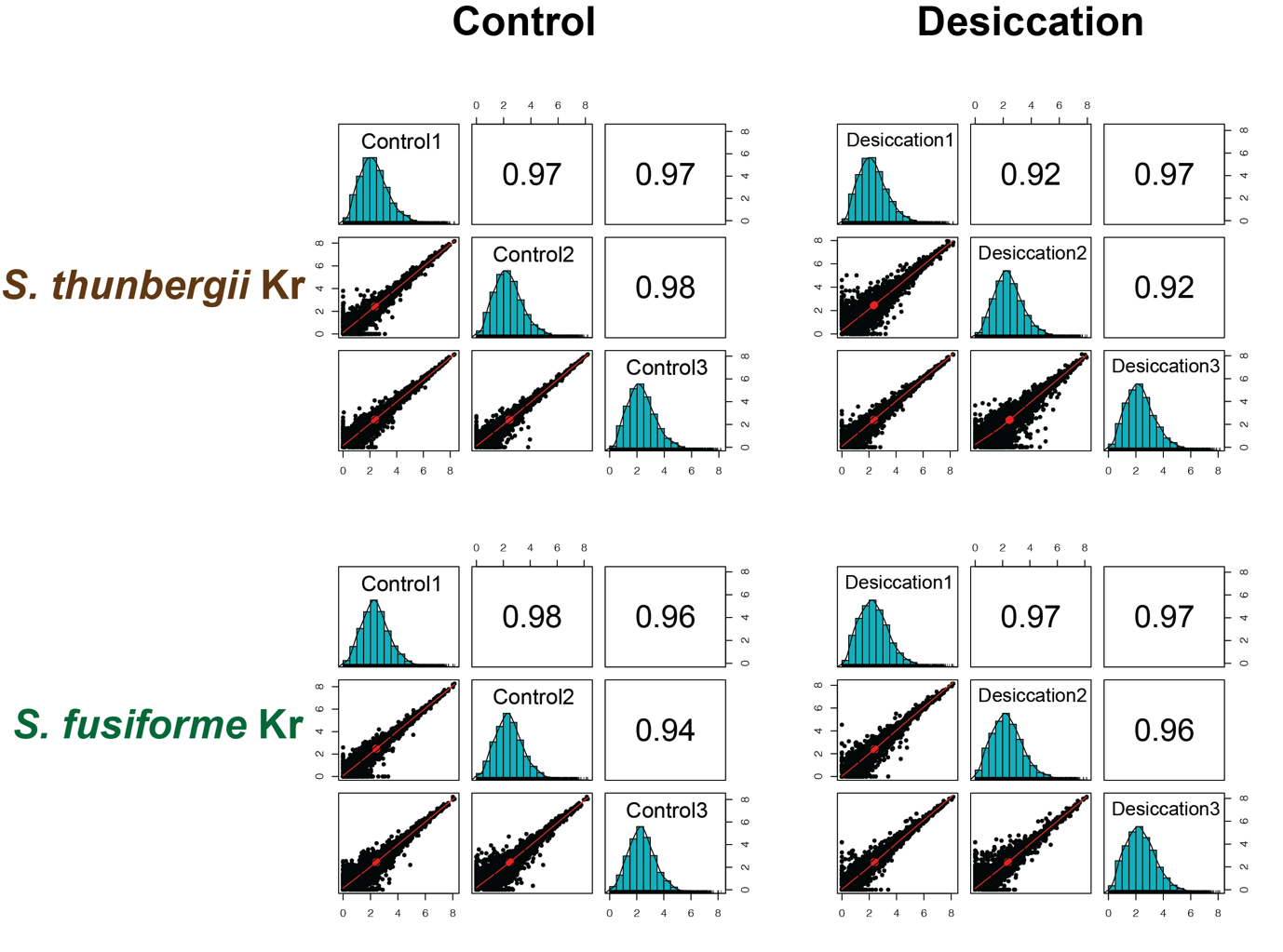


**Figure S15.** Correlation of transcriptome datasets of *S. thunbergii* **and *S.*** *fusiforme* against desiccation stress.

**Supplementary reference**

Armbrust EV, Berges JA, Bowler C, Green BR, Martinez D, Putnam NH, Zhou S, Allen AE, Apt KE, Bechner M, et al. The Genome of the Diatom *Thalassiosira Pseudonana*: Ecology, Evolution, and Metabolism. *Science*. 2004:306:79-86. 10.1126/science.1101156.

Bowler C, Allen A, Badger J, Grimwood J, Jabbari K, Kuo A, Maheswari U, Martens C, Maumus F, Otillar RP, et al. The *Phaeodactylum* genome reveals the evolutionary history of diatom genomes. *Nature*. 2008:456:239-244. 10.1038/nature07410.

Collén J, Porcel B, Carré W, Ball SG, Chaparro C, Tonon T, Barbeyron T, Michel G, Noel B, Valentin K, et al. Genome structure and metabolic features in the red seaweed *Chondrus crispus* shed light on evolution of the Archaeplastida, *Proc. Natl. Acad. Sci.* 2013:110(13):5247-5252. 10.1073/pnas.1221259110.

Derelle E, Ferraz C, Rombauts S, Rouzé P, Worden AZ, Robbens S, Partensky F, Degroeve S, Echeynié S, Cooke R, et al. Genome analysis of the smallest free-living eukaryote *Ostreococcus tauri* unveils many unique features, *Proc. Natl. Acad. Sci.* 2006:103(31):11647-11652. 10.1073/pnas.0604795103.

Fletcher K, Shin OH, Clark KJ, Feng C, Putman AI, Correll JC, Klosterman SJ, Deynze AV, Michelmore RW. Ancestral Chromosomes for Family Peronosporaceae Inferred from a Telomere-to-Telomere Genome Assembly of *Peronospora effusa*. *Mol. Plant-Microbe Interact.*2022:35:450-463. 10.1094/MPMI-09-21-0227-R.

Gobler CJ, Berry DL, Dyhrman ST, Wilhelm SW, Salamov A, Lobanov AV, Zhang Y, Collier JL, Wurch LL, Kustka AB, et al. 2011. Niche of harmful alga *Aureococcus anophagefferens* revealed through ecogenomics. *Proc Natl Acad Sci*. 2011:108:4352-4357. 10.1073/pnas.1016106108.

Kawahara Y, de la Bastide M, Hamilton JP, Kanamori H, McCombie WR, Ouyang S, Schwartz DC, Tanaka T, Wu J, Zhou S, et al. Improvement of the *Oryza sativa* Nipponbare reference genome using next generation sequence and optical map data. *Rice*. 2013:6:4 10.1186/1939-8433-6-4.

Lee JM, Yang EC, Graf L, Yang JH, Qiu H, Zelzion U, Chan CX, Stephens TG, Weber APM, Boo GH, et al. Analysis of the Draft Genome of the Red Seaweed *Gracilariopsis chorda* Provides Insights into Genome Size Evolution in Rhodophyta. *Mol. Biol. Evol*. 2018:35(8):1869–1886. 10.1093/molbev/msy081.

Mahan KM, Polle JEW, McKie-Krisberg Z, Lipzen A, Kuo A, Grigoriev IV, Lane TW, Davis AK. Annotated Genome Sequence of the High-Biomass-Producing Yellow-Green Alga *Tribonema minus*. Microbiol Resour Announc. 2021:10(24) 10.1128/mra.00327-00321.

Matsuzaki M, Misumi O, Shin-i T, Maruyama S, Takahara M, Miyagishima S, Mori T, Nishida K, Yagisawa F, Nishida K, et al. Genome sequence of the ultrasmall unicellular red alga *Cyanidioschyzon merolae* 10D. *Nature.* 2004:428:653–657. 10.1038/nature02398.

Merchant SS, Prochnik S, Vallon O, Harris EH, Karpowicz SJ, Witman GB, Terry A, Salamov A, Fritz-Laylin LK, Maréchal-Drouard L, et al. The *Chlamydomonas* Genome Reveals the Evolution of Key Animal and Plant Functions. *Science.* 2007:318:245-250. 10.1126/science.1143609.

Misner I, Blouin N, Leonard G, Richards TA, Lane CE. The Secreted Proteins of *Achlya hypogyna* and *Thraustotheca clavata* Identify the Ancestral Oomycete Secretome and Reveal Gene Acquisitions by Horizontal Gene Transfer. *Genome Biol. Evol.* 2015:7:120-135. 10.1093/gbe/evu276.

Mock T, Otillar RP, Strauss J, McMullan M, Paajanen P, Schmutz J, Salamov A, Sanges R, Toseland A, Ward BJ, et al. Evolutionary genomics of the cold-adapted diatom *Fragilariopsis cylindrus*. *Nature*. 2017:541:536-540. 10.1038/nature20803.

Radakovits R, Jinkerson R, Fuerstenberg S, Tae H, Settlage RE, Boore JL, Posewitz MC. Draft genome sequence and genetic transformation of the oleaginous alga *Nannochloropsis gaditana*. *Nat. Commun*. 2012:3:686. 10.1038/ncomms1688.

The Arabidopsis Genome Initiative. Analysis of the genome sequence of the flowering plant *Arabidopsis thaliana*. *Nature* 2000:408:796–815. 10.1038/35048692.
